# MicroRNA-181a-5p and microRNA-181a-3p cooperatively restrict vascular inflammation and atherosclerosis

**DOI:** 10.1038/s41419-019-1599-9

**Published:** 2019-05-07

**Authors:** Yingxue Su, Jiani Yuan, Feiran Zhang, Qingqing Lei, Tingting Zhang, Kai Li, Jiawei Guo, Yu Hong, Guolong Bu, Xiaofei Lv, Sijia Liang, Jingsong Ou, Jiaguo Zhou, Bin Luo, Jinyan Shang

**Affiliations:** 1 State Key Laboratory of Ophthalmology, Zhongshan Ophthalmic Center, Sun Yat-sen University, 510060 Guangzhou, China; 2 Department of Pharmacology, Cardiac and Cerebrovascular Research Center, Zhongshan School of Medicine, 510080 Guangzhou, China; 3 Division of Cardiac Surgery, The First Affiliated Hospital, 510080 Guangzhou, China; 4 Guangdong Province Key Laboratory of Brain Function and Disease, Zhongshan School of Medicine, 510080 Guangzhou, China; 5 Program of Kidney and Cardiovascular Disease, The Fifth Affiliated Hospital, 510080 Guangzhou, China; 6 Department of Cardiology, Sun Yat-sen Memorial Hospital, 510080 Guangzhou, China; 7 Department of Forensic Medicine, Zhongshan School of Medicine, Sun Yat-Sen University, 510080 Guangzhou, China

**Keywords:** Health sciences, Atherosclerosis

## Abstract

MicroRNAs have emerged as important post-transcriptional regulators of gene expression and are involved in diverse diseases and cellular process. Decreased expression of miR-181a has been observed in the patients with coronary artery disease, but its function and mechanism in atherogenesis is not clear. This study was designed to determine the roles of miR-181a-5p, as well as its passenger strand, miR-181a-3p, in vascular inflammation and atherogenesis. We found that the levels of both miR-181a-5p and miR-181a-3p are decreased in the aorta plaque and plasma of apoE^−/−^ mice in response to hyperlipidemia and in the plasma of patients with coronary artery disease. Rescue of miR-181a-5p and miR-181a-3p significantly retards atherosclerotic plaque formation in apoE^−/−^ mice. MiR-181a-5p and miR-181a-3p have no effect on lipid metabolism but decrease proinflammatory gene expression and the infiltration of macrophage, leukocyte and T cell into the lesions. In addition, gain-of-function and loss-of-function experiments show that miR-181a-5p and miR-181a-3p inhibit adhesion molecule expression in HUVECs and monocytes-endothelial cell interaction. MiR-181a-5p and miR-181a-3p cooperatively receded endothelium inflammation compared with single miRNA strand. Mechanistically, miR-181a-5p and miR-181a-3p prevent endothelial cell activation through blockade of NF-κB signaling pathway by targeting TAB2 and NEMO, respectively. In conclusion, these findings suggest that miR-181a-5p and miR-181a-3p are both antiatherogenic miRNAs. MiR-181a-5p and miR-181a-3p mimetics retard atherosclerosis progression through blocking NF-κB activation and vascular inflammation by targeting TAB2 and NEMO, respectively. Therefore, restoration of miR-181a-5p and miR-181a-3p may represent a novel therapeutic approach to manage atherosclerosis.

## Introduction

Atherosclerosis is one of the major cause of cardiovascular diseases. The formation and development of atherosclerotic lesions is recognized as a hyperlipidemia-induced chronic inflammatory process involving complex interactions of modified lipoproteins, monocytes and T lymphocytes with cellular components in the vessel wall^1–3^. Vascular inflammation not only drives atherosclerotic plaque development but also contributes to plaque vulnerability^1,3^. Therefore, anti-inflammatory treatment may provide fruitful strategys to prevent atherosclerosis progression.

MicroRNAs (miRNAs) are a class of small, noncoding RNAs that negatively regulate gene expression by targeting the 3′ UTR of specific messenger RNAs (mRNAs) through induction of mRNA degradation or translational repression^4,5^. Dysregulated expression of several proathergenic and antiatherogenic miRNAs has been recently characterized as important mechanisms for atherosclerosis development^6–9^. MiR-33a/b, miR-19b and miR-144-3p have been demonstrated to be involved in regulation of lipid metabolism and/or modulation of cholesterol efflux, and their pharmacological inhibition retarded atherosclerotic lesion development^10–16^. MiR-92a, miR-155, and miR-342-5p have been shown to be critical regulators of inflammation and inhibition of these miRNAs also lead to reduced atherosclerotic lesional size^17–20^. In the contrast, miR-30c, miR-126-5p, and miR-181b have been identified as atheroprotective miRNAs and overexpression of these miRNAs remarkably prevent atherosclerosis through controlling lipid synthase, endothelial cell repair and vascular inflammation^21–23^. These findings indicated that miRNAs are essential regulators of lipid metabolism, inflammation and atherogenesis. Targeting miRNAs may be a potential approach to relieving the development of atherosclerosis.

Recently, miR-181a was shown to be an essential regulator of inflammation in macrophages and dendritic cells^24^. Of note, miR-181a expression is significantly decreased in monocytes from obese patients and patients with coronary artery disease (CAD)^25^. A downregulation of miR-181a has also been observed in the aortic intima of apoE^−/−^ mice received high-fat diet (HFD)^23^. These data suggested that miR-181a might play a critical role in atherosclerosis. However, to date there is no direct evidence to support the involvement of miR-181a in atherosclerosis development. Here, we provide the evidence that both miR-181a-5p and miR-181a-3p are antiatherogenic miRNAs, miR-181a-5p, and miR-181a-3p that mimics and restrict the development of atherosclerotic lesions through inhibition of vascular inflammation by targeting NF-κB signaling pathway.

## Results

### MiR-181a-5p and miR-181a-3p expression are both decreased in atherosclerotic plaque and plasma of apoE^−/−^ mice and plasma of CAD patients

**Fig. 1:**
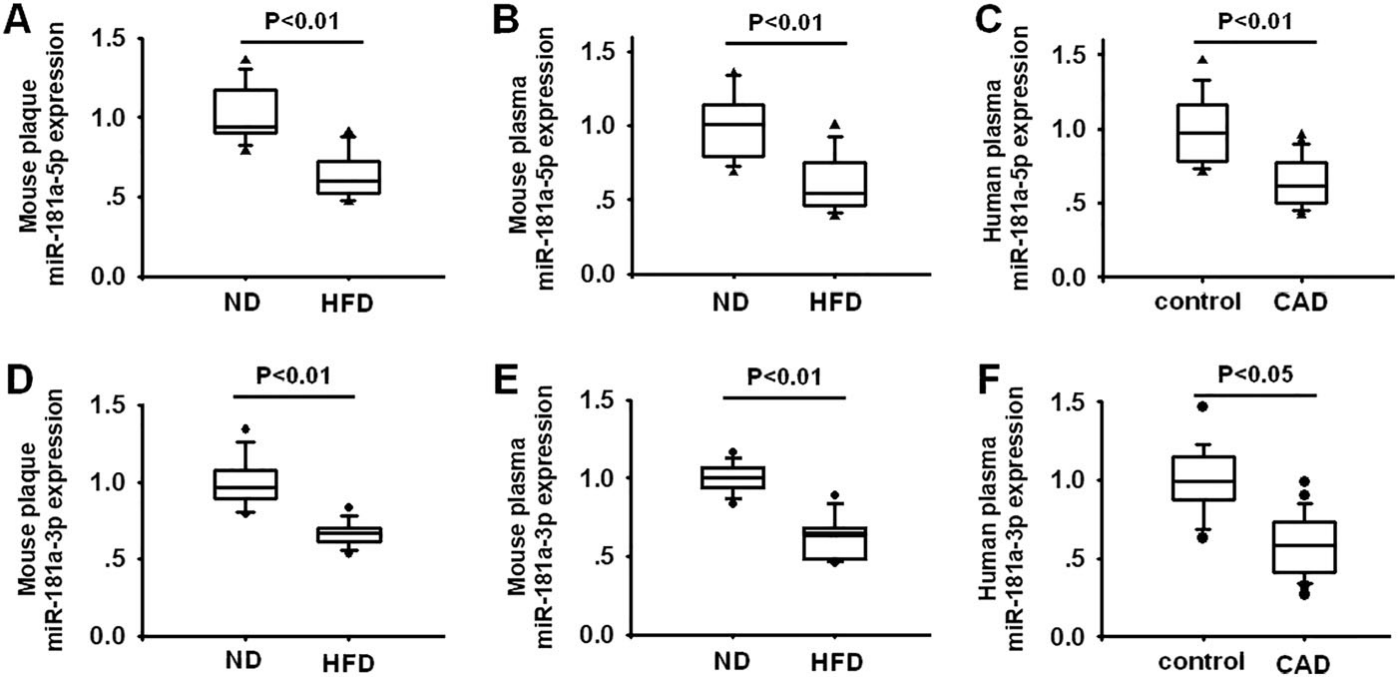
Decreased expression of miR-181a-5p and miR-181a-3p in aorta plaque and plasma of apoE^−/−^ mice fed a high-fat diet (HFD) and plasma of patients with CAD. **a**, **d** miR-181a-5p and miR-181a-3p expression were examined by quantitative polymerase chain reaction (qPCR) in the aorta plaque from apoE^−/−^ mice fed a normal diet (ND) or HFD for 12 weeks (*n* = 10). **b**, **e** plasma miR-181a-5p and miR-181a-3p expression was detected by qPCR in apoE^−/−^ mice fed ND or HFD for 8 weeks (*n* = 10). **c**, **f** circulating miR-181a-5p and miR-181a-3p expression were determined by qPCR in human plasma samples from human subjects without (*n* = 15) or with CAD (*n* = 20)

The precursor miR-181a (pre-miR-181a) can be processed intracellularly to form two mature strands, miR-181a-5p and miR-181a-3p. MicroRNA expression is simultaneously dysregulated in disease state. A study previously demonstrate that miR-181a expression is reduced in aortic intima harvested from ApoE^−/−^ mice fed a HFD^23^. This indicated that miR-181a may be associated with regulating vascular function and atherogenesis. To verify the expression of miR-181a during atherosclerosis, we first examined miR-181a-5p, as well as miR-181a-3p levels in aortic plaque and plasma of apoE^−/−^ mice fed a high fat diet (HFD). As shown in Fig. 1, miR-181a-5p and miR-181a-3p expression in aortic plaque are both remarkably decreased in apoE^−/−^ mice after 8 weeks of HFD (Fig. 1a, d). In addition, plasma circulating miR-181a-5p and miR-181a-3p levels are also remarkably reduced in HFD fed apoE^−/−^ mice (Fig. 1b, e). Further, consistent with the results from animal studies, the levels of miR-181a-5p and miR-181a-3p in plasma collected from CAD patients are much lower than that from healthy controls (Fig. 1c, f). These findings suggest downregulation of miR-181a-5p and miR-181a-3p may contribute to the development of atherosclerosis.

### MiR-181a-5p and miR-181a-3p limit atherosclerosis development in apoE^−/−^ mice

**Fig. 2:**
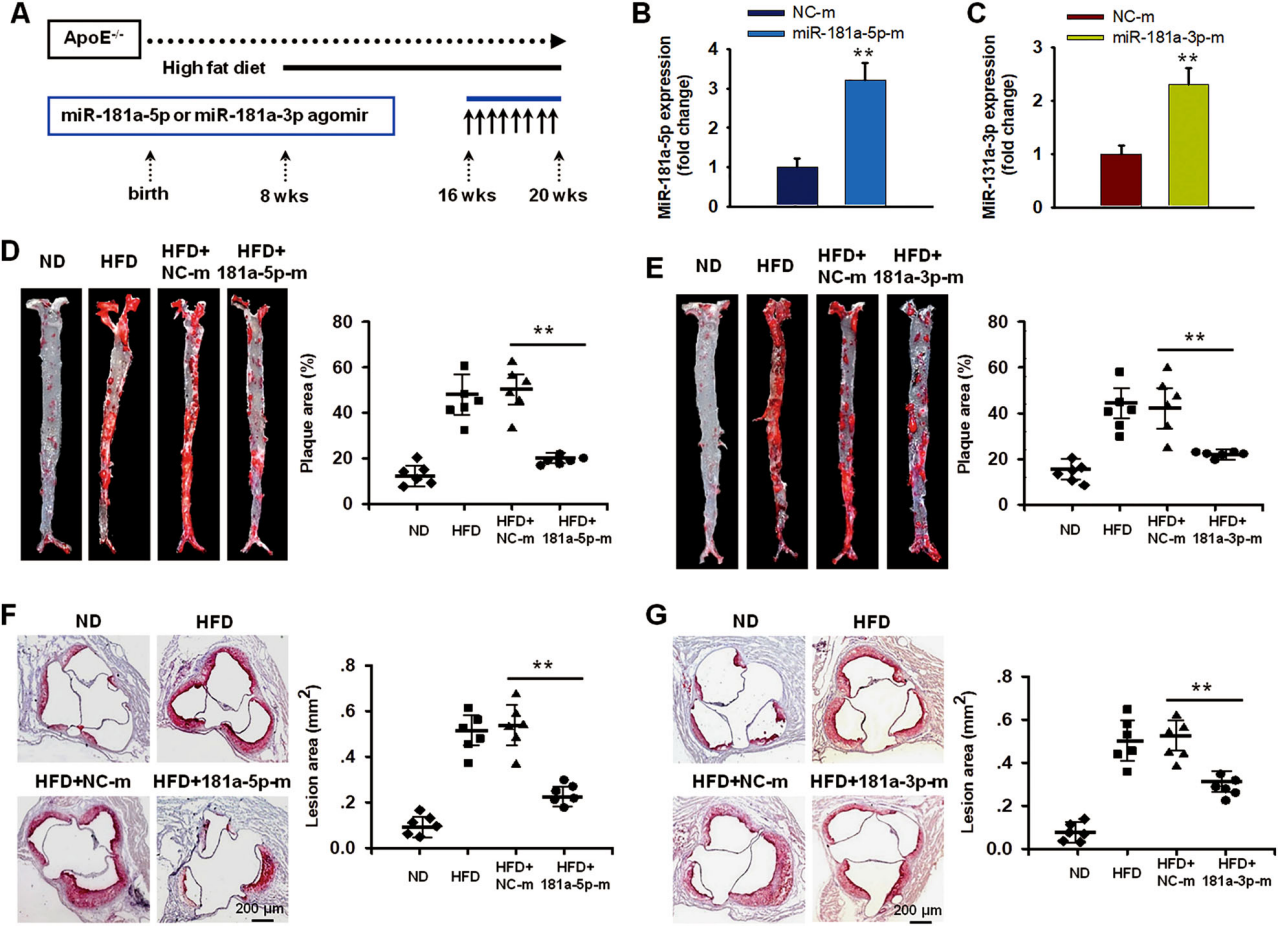
Restoration of miR-181a-5p and miR-181a-3p reduces atherosclerotic lesions in apoE^-/−^ mice. **a** Schema of experimental design. **b**, **c** miR-181a-5p (**b**) or miR-181a-3p (**c**) expression in aorta was detected by qPCR in apoE^−/−^ mice received nonspecific control miRNA (NC-m), miR-181a-5p or miR-181a-3p mimics (*n* = 5). **d**, **e** plaque sizes were quantified using Oil-red O staining in the aorta of apoE^−/−^ mice received NC-m, miR-181a-5p (**d**) or miR-181a-3p (**e**) mimics. The lesion areas were quantified as a percentage of the total aortic surface (*n* = 6). **f**, **g** quantification of lesion areas using Oil-red O staining in the aortic sinus of apoE^−/−^ mice received NC-m, miR-181a-5p (**f**) or miR-181a-3p mimics (**g**) (*n* = 6). **P < 0.01

Above envidence uncovered that miR-181a-5p and miR-181a-3p are reduced during atherosclerosis. To explore the effects and mechanism of miR-181a-5p and miR-181a-3p on atherosclerosis, we proceeded systemic delivery of miR-181a-5p and miR-181a-3p mimics by tail vein injection and examined the effects on atherosclerotic plaque size. Eight-week old apoE^−/−^ mice were fed with hight-cholesterol diet for 8 weeks and the mimics were introduced for 4 weeks (Fig. 2a). MiR-181a-5p and miR-181a-3p expression in aorta intima from mice injected miR-181a-5p and miR-181a-3p mimics were 3.1-fold and 2.3-fold higher than that in mice injected control mimics, respectively (Fig. 2b, c), but these mimics did not affect miR-181b and miR-181c expression in the aorta (Supplementary Fig. S1). In miR-181a-5p overexpressed group, the atherosclerotic plaque area in thoracic and abdominal aorta was significantly decreased (19.1 ± 0.7% vs. 49.8 ± 3.6%, *p* < 0.01, *n* = 6 in each group) compared with that in control mimics treated mice (Fig. 2d). Consistently, the atherosclerotic plaque size in the aortic sinus was also dramatically alleviated in miR-181a-5p mimics treated group (0.24 ± 0.02 mm^2^ vs. 0.53 ± 0.04 mm^2^, *p* < 0.01, *n* = 6 in each group) (Fig. 2f). Similarly, the results from miR-181a-3p mimics treatment also demonstrated that the atherosclerotic plaque area in aorta and in aortic sinus were remarkably reduced compared with that in control miRNA treated mice (Fig. 2e, g). These demonstrate that miR-181a-5p and miR-181a-3p are protectors in atherosclerotic development.

### MiR-181a-5p and miR-181a-3p alleviate vascular inflammation and myeloid cell recruitment

**Fig. 3:**
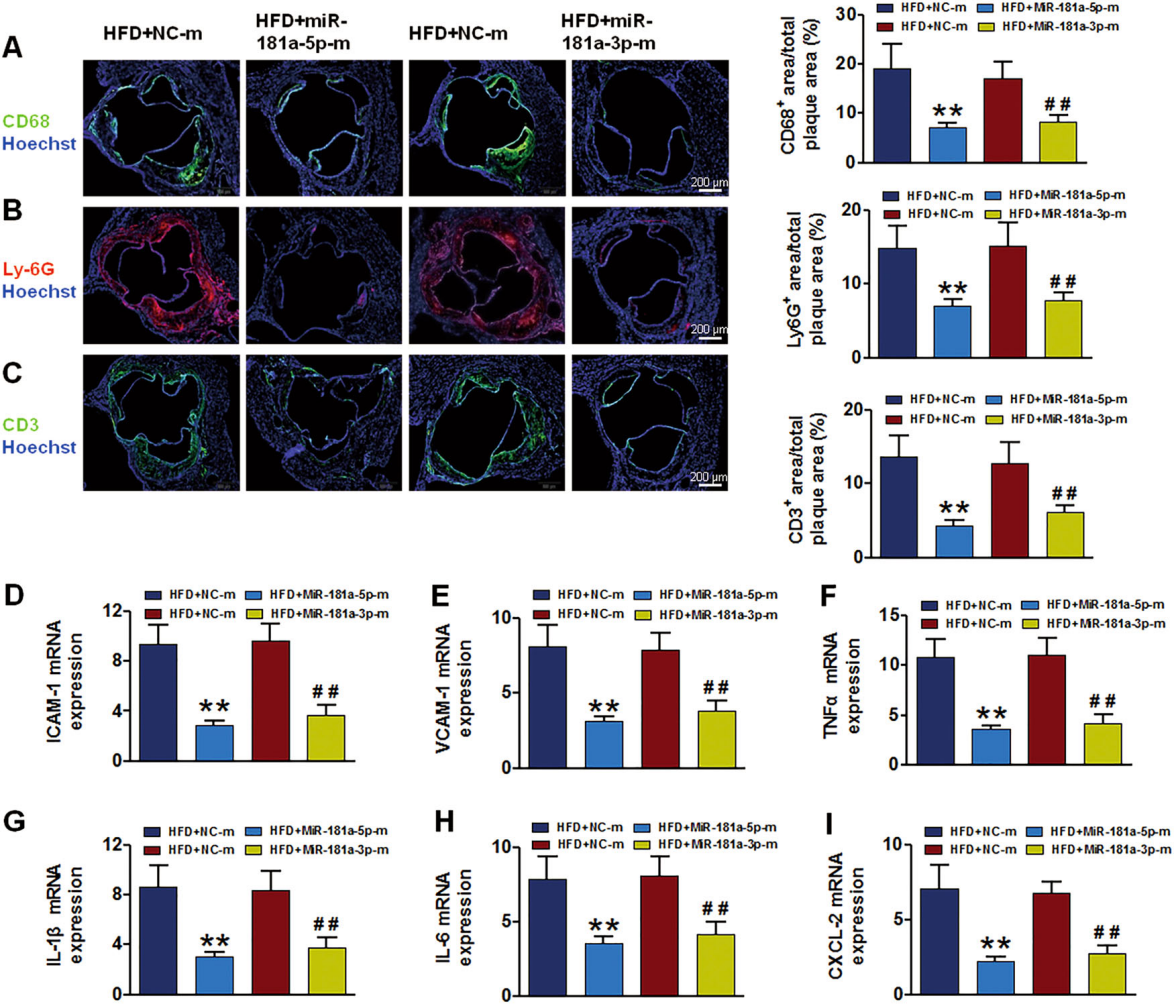
MiR-181a-5p and miR-181a-3p mimics prevent myeloid cell recruitment and vessel inflammation. **a**–**c** representative images and quantification of CD68-positive macrophages (**a**), Ly6G-positive neutrophils (**b**), and CD3-positive T cells (**c**) in the aortic root lesions of apoE^−/−^ mice received NC-m, miR-181a-5p or miR-181a-3p mimics. CD68-positive, Ly6G-positive, and CD3-positive areas are shown as a percentage of total lesion area. **d**–**e** qPCR analysis of ICAM-1 and VCAM-1 mRNA expression in the aortic arch intima. **f**–**i** qPCR analysis of TNF-α (**f**), IL-1β (**g**), IL-6 (**h**) and CXCL2 (**i**) mRNA levels in aorta arch intima of apoE^−/−^ mice treated with nonspecific control miRNA (NC-m), miR-181a-5p or miR-181a-3p mimics. Values are mean ± SEM, *n* = 6 per group, **P < 0.01, ^##^*P* < 0.01 vs. HFD + NC-m, respectively

To investigate the mechanisms how miR-181a-5p and miR-181a-3p prevent atherogenesis, we firstly analyzed the lipid profile in serum. Introduction of miR-181a-5p and miR-181a-3p had no effects on body weight, total cholesterol, triglyceride, low-density lipoprotein, and high-density lipoprotein levels in HFD fed apoE^−/−^ mice compared with controls (Table S1 and S2). Since atherosclerosis is not only a lipid-driven disease, but also a chronic low-grade inflammatory disease of the vessel wall. Vascular inflammation plays a critical role in the initiation and progression of atherosclerosis. Immunofluorescent analysis of atherosclerotic plaques at the aortic sinus of apoE^−/−^ mice received miR-181a-5p and miR-181a-3p mimics showed 62 and 54% reduction of CD68^+^ macrophages (Fig. 3a), 49 and 46% reduction of LyG6^+^ neutrophils (Fig. 3b), and 61 and 48% reduction of CD3^+^ T cells (Fig. 3c), respectively. Vascular inflammation is accompanied by elavated levels of the adhesion molecule and biomarkers of inflammation. In order to assess vascular inflammation upon high-fat diet-induced atherosclerosis in ApoE^−/−^ mice, a panel of adhesion molecules and proinflammatory cytokines were analyzed in aortic arch. As expected, miR-181a-5p and miR-181a-3p mimics administration reduced intercellular cell adhesion molecule-1 (ICAM-1) and vascular cell adhesion molecule-1 (VCAM-1) expression in aortic arch intima by 72 and 61, 64, and 57%, respectively, compared with control miRNA treated apoE^−/−^ mice (Fig. 3d, e). Correspondingly, RT-PCR studies revealed that miR-181a-5p and miR-181a-3p treatment decreased the expression of proinflammatory cytokines and chemokines, including TNF-α, IL-β, IL-6, and CXCL2, in the aortic arch intima of apoE^−/−^ mice (Fig. 3f–i). These data indicate that reduction of vascular inflammation and myeloid cell accumulation to vascular wall rather than lipid metabolism is associated with the benefits of miR-181a-5p and miR-181a-3p on atherosclerotic lesion formation in apoE^−/−^ mice.

### MiR-181a-5p inhibits TNF-α–induced inflammatory response in HUVECs

**Fig. 4:**
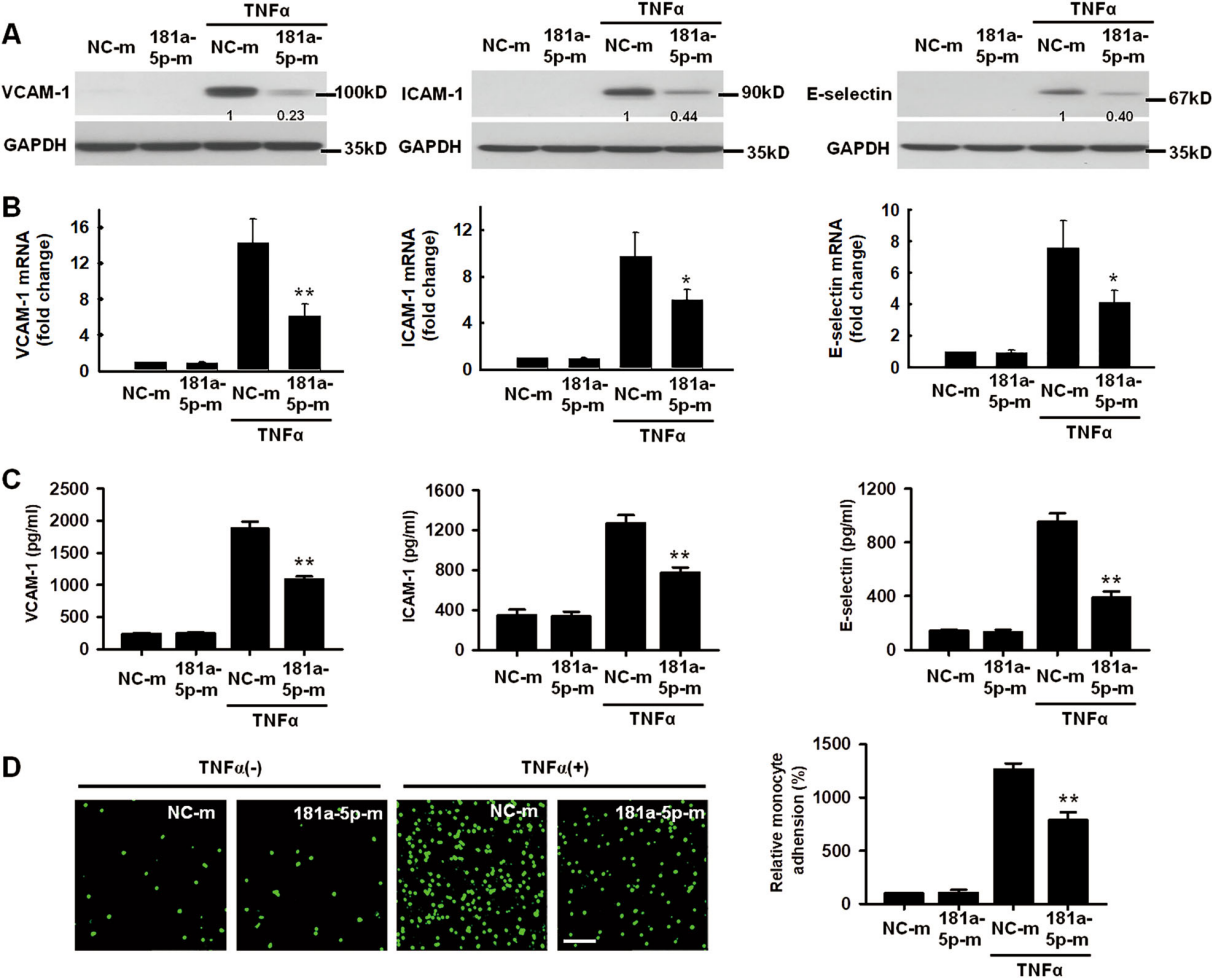
MiR-181a-5p inhibits TNF-α–induced proinfammatory gene expression in HUVECs. **a** VCAM-1, ICAM-1 and E-selectin protein levels in HUVECs transfected with NC-m or miR-181a-5p mimics (181a-5p-m) after TNF-α (10 ng/ml) treatment for 12 h (*n* = 6). **b** VCAM-1, ICAM-1 and E-selectin mRNA levels in HUVECs treated with NC-m or 181a-5p-m after exposure to TNF-α for 4 h (*n* = 7). **c** HUVECs reveived NC-m or 181a-5p-m were exposed to TNF-α for 24 h. VCAM-1, ICAM-1 and E-selectin protein levels in cell culture medium were determined by ELISA analysis (*n* = 6). **d** representative photo images and quantification of calcein-labeled THP-1 monocytes adhering to TNF-α-activated HUVECs transfected with NC-m or 181a-5p-m (*n* = 6). Scale bar: 100 um. *P < 0.05; **P < 0.01 vs. TNF-α + NC-m

To further confirm the role of miR181a-5p in vascular endothelium inflammation, we analyzed the effects of miR-181a-5p on TNF-α-induced inflammatory response in human umbilical vein endothelial cells (HUVECs) by using gain-of-function and loss-of-function approaches. As shown in Fig. 4, miR-181a-5p mimics inhibited TNF-α-induced VCAM-1, ICAM-1, and E-selectin protein expression by 48, 44, and 40%, respectively (Fig. 4a). In contrast, miR-181a-5p inhibitors increased TNF-α-induced VCAM-1, ICAM-1, and E-selectin protein expression by 53, 64, and 39%, respectively (Supplementary Fig. S2a). In line with the protein experiments, TNF-α-induced mRNA levels of VCAM-1, ICAM-1, and E-selectin were also decreased or increased after introduction of miR-181a-5p mimics or miR-181a-5p inhibitors (Fig. 4b and Supplementary Fig. S2b). Consistently, ELISA analysis showed that miR-181a-5p mimics reduced, while miR-181a-5p inhibitors enhanced, the levels of soluble VCAM-1, ICAM-1, and E-selectin in the cell culture medium (Fig. 4c and Supplementary Fig. S2c). The inhibitory effects of miR-181a-5p mimics on VCAM-1 and ICAM-1 protein expression in HUVECs were also observed after oxLDL and LPS treatment (Supplementary Fig. S3a, b). Since adhesion of monocytes to the endothelium is essential factors to initiate and promote endothelium inflammation, we next performed in vitro cell adhesion experiments to determine the interaction of monocytes with endothelial cells (ECs). As expected, miR-181a-5p mimics administration significantly reduced the adhesion capability of THP-1 cells to HUVECs (Fig. 4d), whereas miR-181a-5p inhibitors increased their adhesion (Supplementary Fig. S2d). Together, these results suggest that miR-181a-5p is a negative regulator of adhesion molecules expression and thus inhibits the adhesion of monocytes to ECs.

### MiR-181a-3p inhibits TNF-α–induced expression of adhesion molecules in HUVECs

**Fig. 5:**
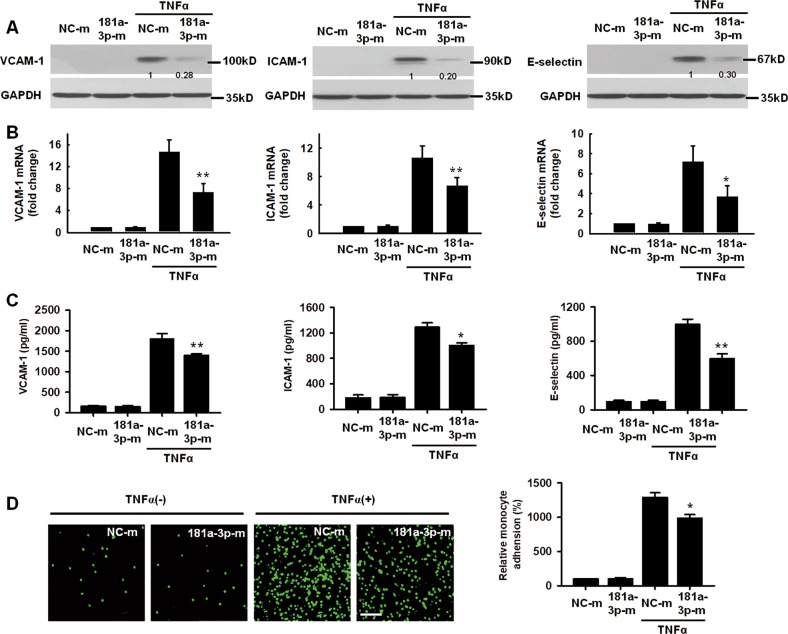
MiR-181a-3p suppresses proinfammatory gene expression in HUVECs. **a** VCAM-1, ICAM-1 and E-selectin protein levels in HUVECs transfected with NC-m or miR-181a-3p mimics (181a-3p-m) after TNF-α (10 ng/ml) treatment for 12 h (*n* = 6). **b** VCAM-1, ICAM-1 and E-selectin mRNA levels in HUVECs treated with NC-m or 181a-3p-m after exposure to TNF-α for 4 h (*n* = 7). **c** HUVECs reveived NC-m or 181a-3p-m were exposed to TNF-α for 24 h. VCAM-1, ICAM-1, and E-selectin protein levels in cell culture medium were determined by ELISA analysis (*n* = 6). **d** representative photo images and quantification of calcein-labeled THP-1 monocytes adhering to TNF-α-activated HUVECs transfected with NC-m or 181a-3p-m (*n* = 6). Scale bar: 100 um. *P < 0.05; **P < 0.01 vs. TNF-α + NC-m

To determine the potential role of miR-181a-3p in regulating inflammatory response, we assessed the effects of miR-181a-3p mimics and its inhibitors on adhesion molecule expression and leukocyte-EC interaction. Introduction of miR-181a-3p mimics in HUVECs decreased TNF-α-induced VCAM-1, ICAM-1, and E-selectin protein expression (Fig. 5a), whereas miR-181a-3p inhibitors increased their expression (Supplementary Fig. S4a). Consistently, incubation of miR-181a-3p mimics inhibited TNF-α-induced mRNA levels of VCAM-1, ICAM-1, and E-selectin in HUVECs. In contrast, miR-181a-3p inhibitors increased their mRNA levels (Fig. 5b and Supplementary Fig. S4b). In agreement with the protein and mRNA experiments, the levels of soluble VCAM-1, ICAM-1, and E-selectin in the cell culture medium, as measured by ELISA analysis, were also reduced or enhanced after introduction of miR-181a-3p mimics or its inhibitors (Fig. 5c and Supplementary Fig. S4c). The inhibitory effects of miR-181a-3p mimics on VCAM-1 and ICAM-1 protein expression in HUVECs were also observed after oxLDL and LPS treatment (Supplementary Fig. S3c and d). Moreover, in vitro cell adhesion experiments showed that miR-181a-3p mimics significantly reduced the adhesion capability of THP-1 cells to HUVECs (Fig. 5d), while miR-181a-3p inhibitors increased their adhesion (Supplementary Fig. S4d). These data indicate that miR-181a-3p is also a critical negative regulator of adhesion molecule expression and leukocyte-EC interaction in the progression of endothelial inflammation.

### MiR-181a-5p and miR-181a-3p both block the NF-κB signaling pathway in HUVECs

**Fig. 6:**
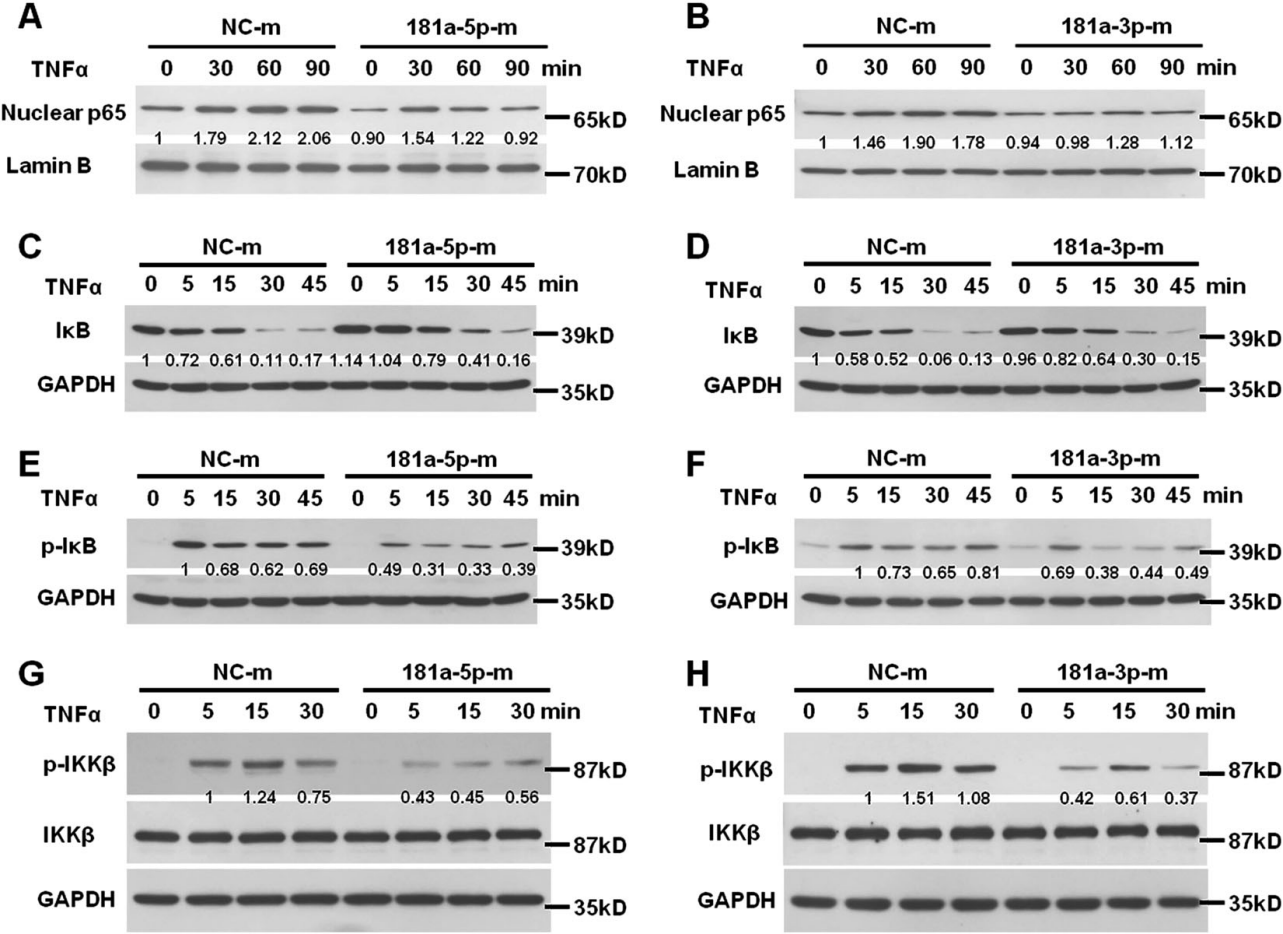
MiR-181a-5p and miR-181a-3p prevent NF-κB activation in HUVECs. **a**, **b** HUVECs received NC-m, 181a-5p-m (**a**) or 181a-3p-m (**b**) were exposed to TNF-α for different times as indicated. The nuclear translocation of p65 was detected by western blotting. c and d, western blotting analysis of IκBα in HUVECs treated with NC-m, 181a-5p-m (**c**) or 181a-3p-m (**d**) followed by TNF-α for different times as indicated. e and f, representative western blotting of phosphorylated IκBα expression in HUVECs transfected with NC-m, 181a-5p-m (**e**) or 181a-3p-m (**f**) followed by TNF-α treatment for different times as indicated. **g**, **h** western blotting analysis of phosphorylated IKKβ in HUVECs treated with NC-m, 181a-5p-m (**g**) or 181a-3p-m (**h**) followed by TNF-α for the indicated times. *N* = 7 per group

Since both miR-181a-5p and miR-181a-3p inhibited adhesion molecule expression and leukocyte-EC interaction in response to different proinflammatory stimuli. Proinflammatory stimulis mediated nuclear factor-κB (NF-κB) activation play a crucial role in the vascular inflammation. We next examined their effects on NF-κB signaling pathway in HUVECs. In the canonical NF-κB signaling pathway, nuclear translocation of NF-κB subunit p65 is an essential step for the activation of NF-κB target proinflammatory genes. Indeed, p65 expression in the nuclear fraction was increased within 30 min after TNFα stimulation (Fig. 6a, b). MiR-181a-5p mimics reduced TNF-α-induced p65 expression in HUVEC nucleus by 41, 53, and 55% after TNF-α treatment for 30 min, 60 min, and 90 min, respectively (Fig. 6a). Likewise, HUVECs received miR-181a-3p mimics also exhibited lower nuclear p65 accumulation triggered by TNF-α than that in HUVECs treated with miRNA negative control (Fig. 6b). Previous studies have shown that importin-α molecules located in nuclear membrane are critical transporters for p65 translocation and miR-181b has been validated to inhibit vascular inflammation by target importin-α3^23^. Our results here demonstrated that neither miR-181a-5p nor miR-181a-3p altered importin-α1 and importin-α3 expression in HUVECs (Supplementary Fig. S5a and b), indicative of the upstream targets underlying the inhibitory effect of miR-181a-5p and miR-181a-3p on p65 nuclear translocation.

Phosphorylation and subsequent proteolysis of IκBα is prerequisite for the release of p65 from IκB complex and its nuclear translocation. To explore the mechanism how miR-181a-5p and miR-181a-3p prevent NF-κB activation, we investigated their effects on TNFα-induced phosphorylation and degradation of IκBα and phosphorylation level of IKKβ. The results showed that TNFα induced IκBα degradation from 15 min and peaked at 30 min (Fig. 6c, d). Compared with miRNA negative control, miR-181a-5p mimics obviously prevented TNF-α-induced IκBα degradation (Fig. 6c). Similar results were also observed in HUVECs exposed to miR-181a-3p mimics (Fig. 6d). The inhibitory effect of miR-181a-5p and miR-181a-3p on IκBα degradation is associated with the reduced phosphorylation levels of IκBα and IKKβ (Fig. 6e–h).

These findings demonstrated that both miR-181a-5p and miR-181a-3p inhibited p65 nuclear translocation through reducing IKKβ and IκBα phosphorylation and subsequent IκBα degradation.

### MiR-181a-3p directly targets NEMO and miR-181a-5p targets TAB2

**Fig. 7:**
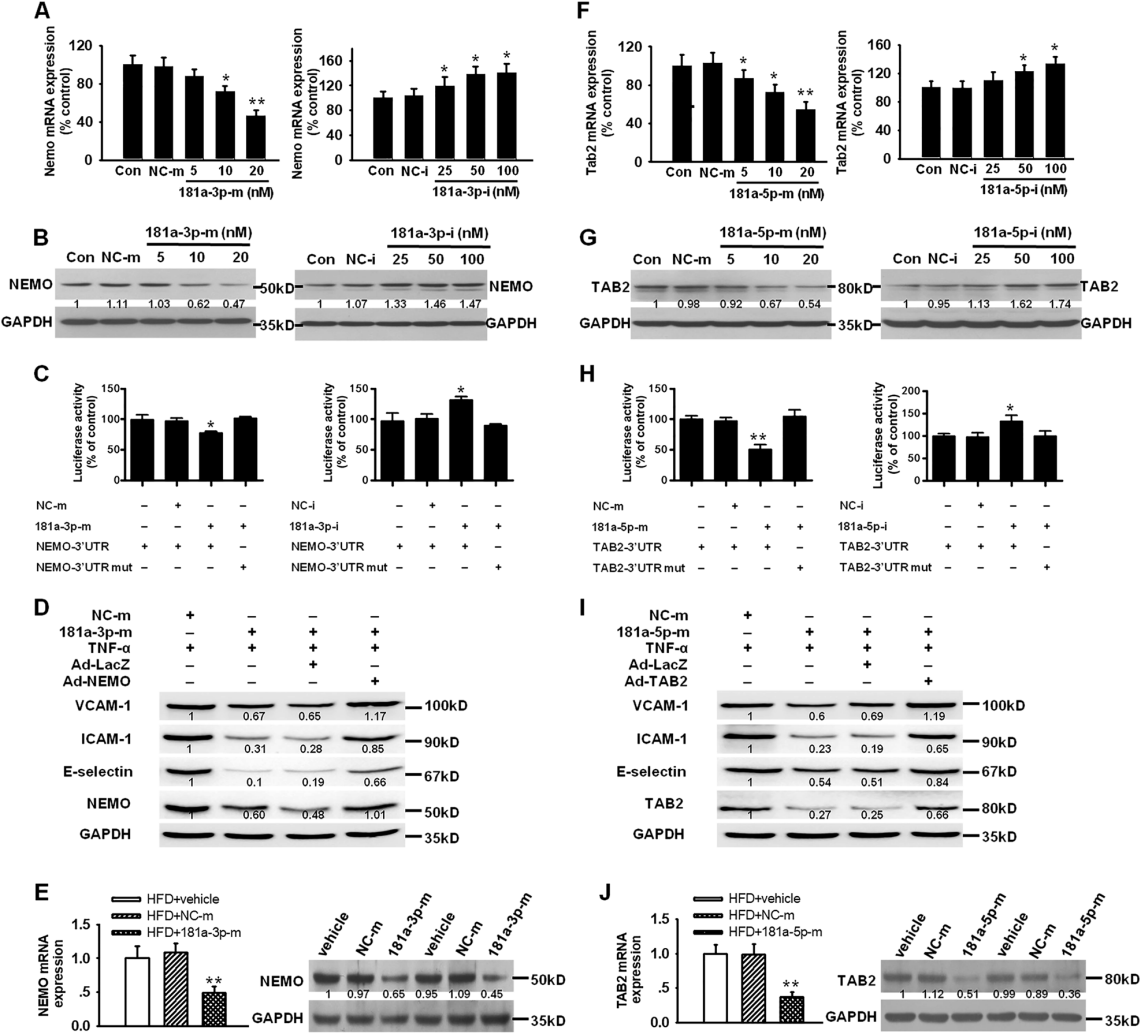
MiR-181a-3p targets NEMO and miR-181a-5p targets TAB2. **a**, **b** NEMO mRNA and protein expression in HUVECs transfected with NC-m or 181a-3p-m, miRNA inhibitor negative control (NC-i) or miR-181a-3p inhibitor (181a-3p-i), *n* = 5. **c** cells were co-transfected with luciferase reporter constructs containing 3′-UTR (Luc-NEMO-3′-UTR) or mutant 3′-UTR (Luc-NEMO-3′-UTR-mut) and with NC-m or 181a-3p-m, NC-i or 181a-3p-i. The luciferase activities were assayed. **d** cells were infected with Ad-LacZ or Ad-NEMO and then were treated with 181a-3p-m or NC-m. The protein levels of VCAM-1, ICAM-1, E-selectin were determined by western blotting. **e** NEMO mRNA and protein levels in aortic intima of apoE^−/−^ mice treated with vehicle, NC-m or miR-181a-3p agomir. **f**, **g** TAB2 mRNA and protein expression in HUVECs transfected with NC-m or 181a-5p-m, NC-i or 181a-5p-i, *n* = 5. **h** luciferase reporter constructs containing 3′-UTR (Luc-TAB2-3′-UTR) or mutant 3′-UTR (Luc-TAB2-3′-UTR-mut) were cotransfected with NC-m or 181a-5p-m, NC-i or 181a-5p-i in cells and the luciferase activities were assayed. **i** cells were infected with Ad-LacZ or Ad-TAB2 and then were treated with 181a-5p-m or NC-m. The protein levels of VCAM-1, ICAM-1, E-selectin were examined. **j** TAB2 mRNA and protein levels in aortic intima of apoE^−/−^ mice treated with vehicle, NC-m or miR-181a-5p agomir, *n* = 6. *P < 0.05; **P < 0.01 vs. NC-m or NC-i

To explore the molecular mechanisms by which miR-181a-5p and miR-181a-3p blunt NF-κB signaling pathway, we identified the potential targets of miR-181a-5p and miR-181a-3p using publicly available algorithms (TargetScan, miRanda and Pic Tar). Computational analysis predicts NEMO as a potential target for miR-181a-3p. To validate that NEMO is regulated by miR-181a-3p, we transfected HUVECs with miR-181a-3p mimics or inhibitors. The results demonstrated that miR-181a-3p mimics inhibited, whereas its inhibitors increased, NEMO mRNA and protein expression (Fig. 7a, b). However, miR-181a-3p mimics did not affect the levels of IKKα and IKKβ proteins (Supplementary Fig. S5d). To confirm the direct interaction between miR-181a-3p and NEMO mRNA 3′-UTR, we cloned NEMO 3′-UTR luciferase reporter plasmid and performed reporter analysis. As expected, miR-181a-3p mimics repressed, while miR-181a-3p inhibitors enhanced, the luciferase activity (Fig. 7c). But miR-181a-3p mimics failed to repress the activity of NEMO 3′-UTR reporter with a mutated miR-181a-3p seed sequence (Fig. 7c). To elucidate whether inhibition of NEMO underlies the anti-inflammation of miR-181a-3p, the effects of miR-181a-3p mimics on inflammatory responses were examined in HUVECs infected with adenovirus vector harboring NEMO gene (Ad-NEMO) or control adenovirus vector (Ad-LacZ). Our results showed that Ad-NEMO infection reversed 181a-3p mimics induced downregulation of VCAM-1, ICAM-1, and E-selectin expression both in mRNA (Supplementary Fig. 6a) and protein (Fig. 7d) levels in HUVECs, as well as the concentration of soluble VCAM-1, ICAM-1, and E-selectin in the cell culture medium (Supplementary Fig. 6b) and the adhesion of monocytes to HUVECs (Supplementary Fig. 6c). To further determine whether NEMO is a functional target of miR-181a-3p in vivo during atherogenesis, NEMO expression were evaluated in aortic intima. As shown in Fig. 7e, NEMO mRNA and protein expression were obviously reduced in aortic intima of apoE^−/−^ mice received miR-181a-3p mimics compared with non-specific miRNA treated apoE^−/−^ mice. These data established that miR-181a-3p inhibits vascular inflammation and atherogenesis by targeting NEMO.

MiR-181a-5p mimics or inhibitors also decreases or increases NEMO protein expression, but NEMO is not the direct target of miR-181a-5p because its mimics or inhibitor had no effect on NEMO 3′-UTR luciferase activity (Supplementary Fig. S7). MiR-181a-5p mimics also had no effects on IKKα and IKKβ protein expression (Supplementary Fig. S5c). Further analysis identified several upstream effectors including RIP1, CARD11, TAB2, and TAB3 as potential targets for miR-181a-5p. However, miR-181a-5p did not affect the expression of RIP1, CARD11, and TAB3 in HUVECs (Supplementary Fig. S8). Of note, miR-181a-5p mimics remarkably decreased, while its inhibitors significantly increased, TAB2 mRNA and protein expression in HUVECs (Fig. 7f, g). Further studies demonstrated that miR-181a-5p mimics repressed, whereas miR-181a-5p inhibitors enhanced, the activity of TAB2 3′-UTR reporter (Fig. 7h). But miR-181a-5p failed to inhibit the activity of TAB2 3′-UTR reporter with a mutated miR-181a-5p seed sequence (Fig. 7h). Moreover, infection of adenovirus vector harboring TAB2 gene (Ad-TAB2) reversed 181a-5p mimics induced downregulation of VCAM-1, ICAM-1 and E-selectin expression both in mRNA (Supplemental Fig. 6d) and protein (Fig. 7i) levels in HUVECs, as well as the concentration of soluble VCAM-1, ICAM-1, and E-selectin in the cell culture medium (Supplemental Fig. 6e) and the adhesion of monocytes to HUVECs (Supplemental Fig. 6f). Moreover, apoE^−/−^ mice received miR-181a-5p mimics exhibited a markedly decrease of TAB2 mRNA and protein levels in aortic intima compared with non-specific miRNA treated apoE^−/−^ mice (Fig. 7j). These data identified TAB2 as a direct target of miR-181a-5p.

### MiR-181a-5p and MiR-181a-3p cooperatively relieved vascular endothelium inflammation

**Fig. 8:**
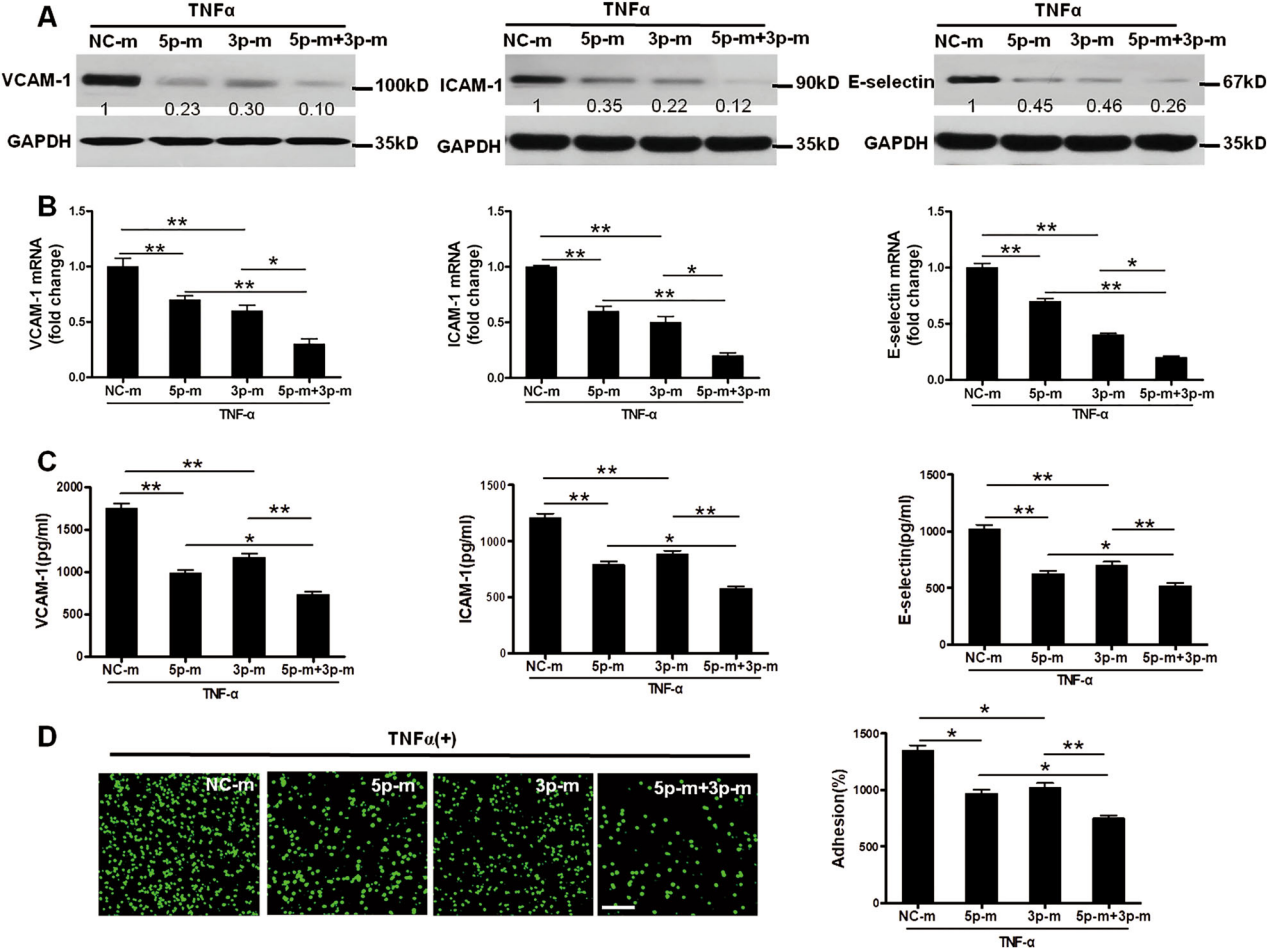
MiR-181a-5p and miR-181a-3p cooperatively inhibits TNF-α-induced proinflammatory gene expression in HUVECs. HUVECs were transfected with NC-m, miR-181a-5p mimics (5p-m), miR-181a-3p mimics (3p-m), respectively, or co-transfected with them in response to TNF-α (10 ng/ml). **a** western blotting analysis of VCAM-1, ICAM-1 and E-selectin in HUVECs after transfection exposure to TNF-α (10 ng/ml) for 12 h (*n* = 6). **b** Real-time quantitative polymerase chain reaction (qPCR) analysis of VCAM-1, ICAM-1 and E-selectin in HUVECs after TNF-α treament for 4 h (*n* = 7). **c** VCAM-1, ICAM-1, and E-selectin protein levels in cell culture medium were determined by ELISA analysis (*n* = 6). **d** representative photo images and quantification of calcein-labeled THP-1 monocytes adhering to TNF-α-activated HUVECs (*n* = 6). Scale bar: 100 um. *P < 0.05; **P < 0.01

MiR-181a-5p and miR-181a-3p are both negative regulators of NF-κB activation in vascular endothelium. We next examined whether co-transfection of miR-181a-5p and miR-181a-3p exerts cooperative anti-inflammatory effect in HUVECs. The results demonstrated that co-transfection of miR-181a-3p and miR-181a-5p mimics in HUVECs exerted more profound inhibitory effects on TNF-α-induced VCAM-1, ICAM-1, and E-selectin expression than single miRNA mimic, both in mRNA and protein levels (Fig. 8a–c). Consistently, simultaneous incubation of miR-181a-3p and miR-181a-5p mimics more significantly reduced the adhesion capability of monocytes to HUVECs (Fig. 8d). These findings highlight that a strong inhibition of miR-181a-5p and miR-181a-3p in the vascular endothelium inflammation and the potencial clinical use to treat chronic vascular inflammatory diseases.

## Discussion

The results presented here identify that miR-181a-5p and its passenger strand miR-181a-3p are antiatherogenic miRNAs. Both miR-181a-5p and miR-181a-3p act as negative post-transcriptional regulators of NF-κB signaling pathway by targeting TAB2 and NEMO, respectively. Restoring miR-181a-5p and miR-181a-3p expression alleviates vascular inflammation in response to hyperlipidemic stress, leading to retard pathological progression of atherosclerosis.

The important role of miRNAs in cardiovascular diseases, including atherosclerosis, has been evidenced by recent observations^7–9,26–28^. MiR-33a/b, miR-19b, miR-144-3p, miR-92a, miR-155, miR-145/143, and miR-342-5p have been identified to promote atherosclerosis development^10–12,15–20,29–31^. In contrast, miR-30c, miR-126-5p, and miR-181b were characterized as inhibitors of atherogenesis^21–23^. MiR-181a belongs to miR-181 family, which includes miR-181a, miR-181b, miR-181c, and miR-181d. Among these, miR-181b has been demonstrated to play a critical role in regulation of vascular inflammation and atherosclerosis progression by reducing the expression of importin-α3 (IPOA3), a protein critical for NF-κB translocation from cytoplasm to nucleus^23,32^. Of note, this study also showed that that miR-181a is the second most dominantly expressed miR-181 family member in the aortic intima. MiR-181a expression is reduced in the aorta intima of apoE^−/−^ mice received HFD^23^. Interestingly, miR-181a was decreased in obese patients. MiR-181a, but not miR-181b and miR-181d, level is lower in CAD patients^25^. These data together imply that miR-181a-5p and miR-181a-3p may be essential regulators for atherosclerosis development. Indeed, our results here provide strong evidence that miR-181a-5p and miR-181a-3p are antiatherogenic miRNAs. MiR-181a-5p and miR-181a-3p mimics limit atherosclerotic lesion progression in apoE^−/−^ mice.

Dyslipidemia is an independent risk factor for atherosclerosis. MiR-33a/b, miR-19b, miR-144-3p, and miR-30c have been identified as critical regulators of lipid metabolism^10–16,22,29^. Our present data reveal that miR-181a overexpression has no effects on plasma total cholesterol, triglyceride, low-density lipoprotein, and high-density lipoprotein levels in HFD fed apoE^−/−^ mice, suggesting other mechanism rather than lipid-lowering effect underlying the anti-atherogenesis of miR-181a.

Since atherosclerosis is not only a lipid-driven disease, but also a chronic low-grade inflammatory disease of the vessel wall, vascular inflammation and activation of immune cells play crucial roles in the pathphysiology of atherosclerosis^33–35^. MiR-181a-5p has been reported to be a essential regulator of T lymphocyte differentiation and activation^36–38^. Overexpression of miR-181a-5p enhances the sensitivity and signaling strength of T lymphocyte in response to antigens^36–38^. However, our results here showed that T cell, leukocyte and macrophage contents in atherosclerotic lesions were virtually reduced in HFD fed apoE^−^^/−^ mice received miR-181a-5p and miR-181a-3p. In addition, miR-181a-5p and miR-181a-3p treatment obviously decreased the expression of adhesion molecules such as ICAM-1 and VCAM-1 in aortic arch and in HUVECs, and inhibited the adhesion of leukocyte to endothelial cells. These findings indicate that defect of adhesive capability after miR-181a-5p and miR-181a-3p mimics treatment is likely the cause of the declined myeloid cell infiltration into the vascular wall.

MiR-181a-5p has been reported to play an important role in inflammation in macrophages and dendritic cells by targeting IL-β and c-Fos^24,39^. However, little is known about the functions of miR-181a-3p. Here, we provide evidence that both miR-181a-5p and miR-181a-3p are critical regulators of vascular inflammation. MiR-181a-5p and miR-181a-3p inhibit vascular inflammation through regulation of NF-κB signaling pathway by targeting TAB2 and NEMO, respectively, uncovering the common mechanism of the inhibitory effects of miR-181a-5p and miR-181a-3p on inflammation in response to different types of proinflammatory stimuli. NEMO is the essential regulatory/scaffold subunit of the IκB kinase (IKK) complex for inflammation stimuli induced canonical NF-κB activation, leading to phosphorylation and degradation of IκBs and then nuclear translocation of p65^40^. It is reported that knockdown of NEMO by RNA interference in Jurkat or human primary T cells blocked inflammation signals such as TNF-α and LPS-induced NF-κB activation by adjusting IκBα degradation to happen later and last shorter^41^. As expect, miR-181a-3p decreased the expression of NEMO in HUVECs, leading to reducing ICAM-1, VCAM-1, E-selectin production by weakening of IκBα degradation and subsequent p65 nuclear translocation. TAB2 is an adapter protein that facilitates TNFα and IL-1β-mediated NF-κB activation^42^. Previous studys show that TAB2 and TAB3, two targets of miR-23b, are essential for autoimmune inflammation signaling mediated by TNF-α or IL-1β^43^. Our present findings demonstrated that miR-181a-5p targeted TAB2 but not TAB3 and subsequently inhibited TNFα triggered NF-κB activation.

**Fig. 9:**
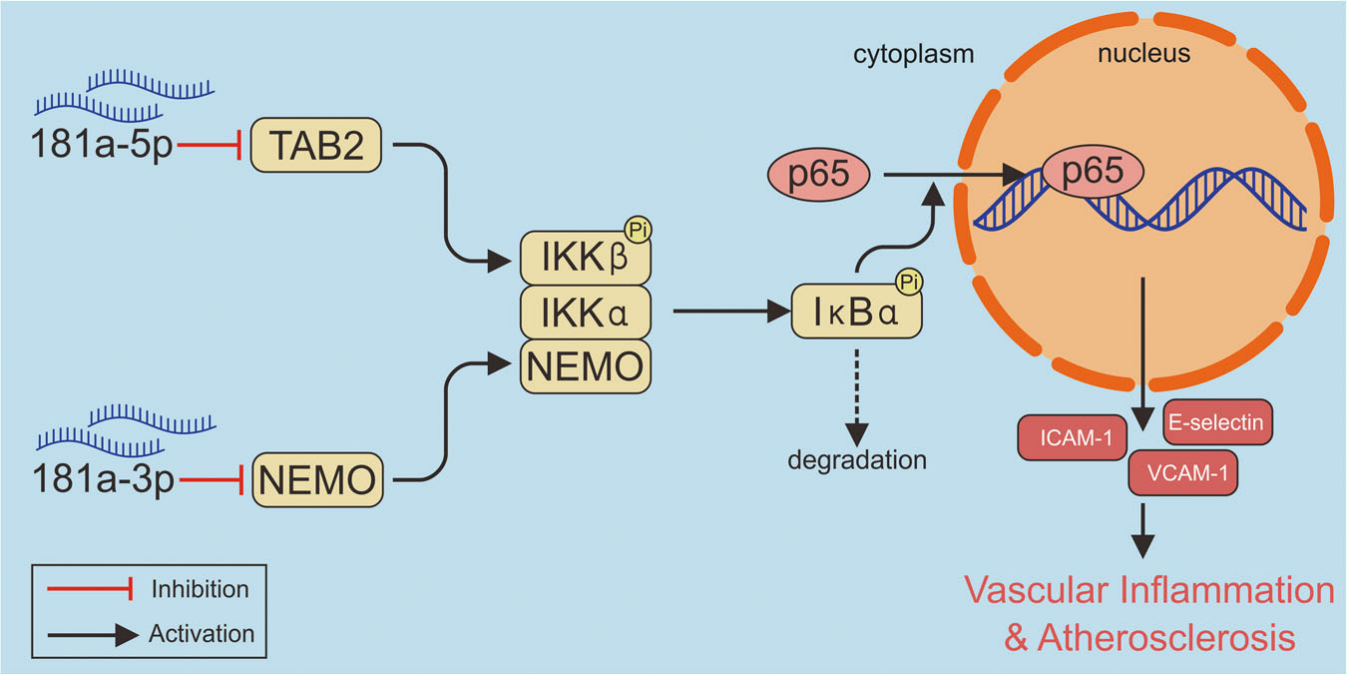
A schematic diagram explaining the anti-atherosclerotic effects of miR-181a

In conclusion, our findings here demonstrate for the first time that miR-181a-5p and its passager strand miR-181a-3p are antiatherogenic miRNAs and exert synergistic effects in vascular endothelium inflammation. MiR-181a-5p and miR-181a-3p mimetics retard atherosclerosis progression through targeting TAB2 and NEMO, respectively, to block NF-κB activation and vascular inflammation (Fig. 9). Accordingly, restoration of miR-181a-5p and miR-181a-3p is likely a novel therapeutic approach for treating atherosclerosis.

## Materials and methods

### Reagents and antibodies

M199 medium, RPMI 1640 medium and fetal calf serum were obtained from Gibco (Grand Island, NY, USA). Tumor necrosis factor α (TNF-α), lipopolysaccharides (LPS), oxidized low density lipoprotein (oxLDL), oil red O, Hoechst 33258 were purchased from Sigma (St.Louis, MO, USA). Antibodies against ICAM-1, VCAM-1, importin-α1, importin-α3, TAB2, TAB3, RIP1, CARD11, IKKα, IKKβ, NEMO, IκBα, p65, phospho-IKKβ and phospho-IκBα were from Cell Signaling Technology (Danvers, MA, USA). E-selectin antibody was get from Proteintech (Chicago, USA). Ly6G, CD68, and CD3 used in our study were purchased from Abcam (Cambrige, England, UK). MiRNA negative control, miR-181a-5p mimic, miR-181a-3p mimic, miRNA inhibitor negative control, miR-181a-5p inhibitor and miR-181a-3p inhibitor were from Genepharma Co. (Shanghai, China). ELISA kit to evaluate ICAM-1, VCAM-1, and E-selectin were from Boster Biological Engineering Co. (Wuhan, China). Lipofectamine^TM^ 2000 reagent was from Invitrogen (Carlsbad, USA).

### Cell culture

Human umbilical vein endothelial cells (HUVECs) were isolated and cultured as previously described^32,44^. In brief, HUVECs were harvested by the umbilical vein digested with 0.125% trypsin with 0.01% EDTA, then the cells were cultured in complete M199 medium which supplemented with 20% fetal calf serum, 100 U/ml penicillin, 100 U/ml streptomycin, 25 U/ml heparin, 2 mM L-glutamine and 5 ng/ml recombinant human endothelial growth factor β (β-ECGF) at 37 °C, 5% CO_2_ atmosphere.

THP-1 cells purchased from American Type Culture Collection (ATCC, Manassas, VA, USA) were cultured in RPMI 1640 culture medium with 10% fetal calf serum, 100 units/ml penicillin and 100 μg/ml streptomycin. Cultures were maintained at 37 °C in a humidified incubator in a 95% O_2_ plus 5% CO_2_ atmosphere.

### Human plasma samples

Human plasma samples were obtained from 15 patients who were diagnosed as coronary artery disease (CAD) under coronary angiography and 20 healthy controls. In this study, the presence of significant CAD was defined at least 70% stenosis in coronary artery, whereas absence of CAD was defined as having no detectable lesions on angiography. Patients with a history of cardiovascular disease, including coronary, peripheral artery, or cerebrovascular disease were excluded from the control group. This study protocol was approved by the Medical Research Ethics Committee of Sun Yat-sen University. Informed consent was obtained from all subjects and the experiments were conducted according to the principles expressed in the Declaration of Helsinki.

### Quantitative real-time PCR

Total RNA was isolated from cells or tissues with TRIzol reagent (Invitrogen, Carlsbad, CA, USA). High-Capacity cDNA Reverse Transcription Kit (Applied Biosystems, Foster City, CA, USA) was used for the reverse transcription of extracted RNA into cDNA according to the manufacturer’s instructions. Real-time PCR was performed using SYBR Green fluorescence (ABI, Foster City, USA) as previously described^32,44,45^. After amplification, the threshold cycle (Ct) was determined. The fold change in expression of each gene was calculated based on the 2^−△△CT^ method with 18 S rRNA as an internal control. The primer sequences were listed as supplementary Table 3 in the Supplemental Materials.

MiRNA expression was quantified using the TaqMan MicroRNA Expression Assay (Applied Biosystems), according to the manufacturer’s protocol. MiRNA expression level was normalized to that of the U43 small nuclear RNA (RNU43).

### Luciferase reporter assay

To detect whether miR-181a-5p and miR-181a-3p would directly target NEMO or TAB2, possible miR-181a-5p and miR-181a-3p binding sites were obtained from a miRNA database (targetscan.org, miRanda.org and Pic Tar.org). The luciferase reporter assay was performed using a construct generated by sub-cloning PCR products amplified from 3′UTR full-length of NEMO, TAB2 in the XhoI and NotI restriction sites of the luciferase reporter vector psiCHECK-2 (Promega, Madison, WI, USA). For the luciferase reporter assay, preconfluent (60 to 70%) cells plated in 24-well plates were transiently cotransfected with 500 ng of each reporter construct (wild-type and mutant NEMO or TAB2 3′-UTR or the psiCHECK-2 vector) and the synthetic nonspecific control miRNA, miR-181a-5p, or miR-181a-3p mimics or their inhibitors using Lipofectamine 2000 reagent (Invitrogen). Firefly and Renilla luciferase activities were analyzed 48 h after transfection by Dual-Luciferase Reporter Assay System (Promega, Madison, WI, USA) according to the manufacturer’s instructions. The values were normalized to firefly luciferase. All transfection experiments were performed at least 5 times.

### Generation of recombinant adenovirus

The adenovirus vector harboring TAB2 gene (Ad-TAB2) or NEMO gene (Ad-NEMO) was purchased from Vigene Biosciences (Shandong, China). Ad-LacZ was used as the control.

### Western blotting

Western blotting was preformed as we previously described^32,44–46^. Briefly, cells were rinsed thrice with ice-cold phosphate-buffered saline and lysed with lysis buffer containing: Tris-HCl 50 mmol/L, NaCl 150 mmol/L, NaN3 0.02%, Nonidet P-40 1%, sodium dodecyl sulfate 0.1%, sodium deoxycholate 0.5 and 1% protease inhibitor cocktail (Merk, USA). The protein content was quantified with BCA kit (Thermo Scientific, USA) and separated by 10% SDS-PAGE, and then transformed to polyvinylidene fluoride (PVDF) membranes (Millipore, Bedford, MA, USA). The membranes were blocked in 5% (W/V) non-fat milk diluted with TBST (Tris–HCl 20 mM, NaCl 150 mM, 0.1% Tween 20, pH 7.5) at room temperature for 1 h, incubated with primary antibody for 1 h at room temperature or over-night at 4 °C, and then incubated with the appropriate secondary horseradish peroxidase-conjugated antibodies including HRP-conjugated anti-rabbit or anti-mouse (1:1000; Cell Signaling Technology) for 1 h at room temperature. Blots were detected using a Pierce ECL Plus Substrate (Thermo Scientific, USA) as described by the manufacturer. The density of target bands were quantified with ImageJ software (National Institutes of Health).

### Animal experiments

ApoE^−/−^ mice (8-weeks-old, 20–25 g, B6.129P2-Apoetm1Unc/J, stock no. 002052) were purchased from Jackson Laboratories (Bar Harbor, ME, USA). Atherosclerosis was induced by feeding apoE^−/−^ mice for 12 weeks with a high-fat western-type diet containing 1.25% of cholesterol (Laboratory Animal Facility of Chinese Academy of Sciences, Shanghai, China). To investigate the role of miR-181a-5p and miR-181a-3p in atherosclerosis development in vivo, an equal volume of atelocollagen and miRNAs was mixed to form complexes according to the manufacturer’s instructions. After feeding high-fat diet (HFD) for 8 weeks, adenovirus expression of negative control, miR-181a-5p mimics or miR-181a-3p mimics (Genepharma Co. Shanghai, China) were injected intravenously to the mice twice a week for the last 4 weeks of a 12-week HFD feeding program. All animal experimental procedures were performed in accordance with the policies of the Sun Yat-Sen University Animal Care and Use Committee and conformed to the “Guide for the Care and Use of Laboratory Animals” of the National Institute of Health in China.

### Analysis of atherosclerotic lesions

At 12 weeks of high-fat diet, mice were anesthetized by intraperitoneal injection of pentobarbital sodium. Mouse hearts were perfused with 20 ml of phos-phate-buffered saline (PBS). The total aorta was dissected from the heart to the iliac bifurcation and the adventitial tissue was cleaned carefully under a dissecting microscope. After fixation with 10% buffered formalin for 24 h. The aorta was opened longitudinally and stained with 0.3% oil red-O for 2 h and then were destained in 78% methanol for 5 min. Plaques were analyzed under the Olympus microscope connected to a digital camera with macro conversion lens. The positive staining area was quantified using Image-Pro Plus 5.0 software. The aoric sinus plaque area of each mouse was obtained by the average of the positive staining areas in six sections from the same animal.

### Lipid profile analysis

Total cholesterol and triglyceride levels were measured using the Infinity™ Cholesterol Reagent and Infinity™ Triglycerides Liquid Stable Reagent (Thermo Scientific) according to the manufacturer’s instructions. HDL-cholesterol and LDL-cholesterol were examined by colorimetric assay using HDL and LDL/VLDL cholesterol Assay Kit (Abcam, Cambrige, England, UK).

### Immunofluorescent staining

Frozen tissue sections were treated in a PBS washing buffer containing 0.1% Triton X-100 for 10 min. The sections were then blocked by 20% rabbit serum for 30 min. After blocking, the sections were incubated with primary antibodies against CD68 or CD3 at 4 °C overnight and then were incubated with FITC-labeled or Cy3-labeled anti-rabbit second antibodies (Jackson Immuno Research Lab) at room temperature for 1 h. To examine the infiltration of neutrophils, slides were incubated with R-phycoerythrin (R-PE)-conjugated mouse Ly-6G specific antibody^47^. The nuclei was stained with Hochest 33342. Slides were subsequently examined with a laser-scanning confocal microscopy (FV500, Olympus, Tokyo, Japan) and images were analyzed with Image-Pro Plus software (Media Cybernetics).

### Cell adhesion assay

The adhesion assay of leukocyte to HUVECs in vitro was performed as described previously^44^. Briefly, THP-1 cells were labeled with 3 μM Calcein-AM (Thermo Scientific, Waltham, MA, USA) for 30 min at 37 °C in 5% CO_2_. HUVECs pretreated with nonspecific control miRNA, miR-181a-5p mimics, or miR-181a-3p mimics, miRNA inhibitor negative control, miR-181a-5p inhibitor or miR-181a-3p inhibitor were plated in 35 mm culture dishes at a density of 2 × 10^5^ cells/ml. After incubation with TNF-α (10 ng/ml) for 24 h. The cells were washed twice with RPMI-1640, then the Calcein-AM labeled monocytes were added to each culture dish for 1 h at 37 °C, 5% CO_2_. Then the dishes were gently washed with prewarmed RPMI-1640 to remove non-adherent cells. This was repeated twice and then 1 ml RPMI-1640 medium was added into each dish and image were captured with a laser confocal scanning microscopy (FV500, Olympus, Tokyo, Japan) with an excitaion wavelength of 485 nm and emission at 530 nm. At least 6 fields randomly selected in each dish were observed.

### ELISA

HUVECs were transfected with control miRNA, miR-181a-5p, or miR-181a-3p mimics or their inhibitors. Twenty-four hours later, cells were treated with 10 ng/ml TNF-α for 24 h. The cultured medium were then collected and ICAM-1, VCAM-1, and E-selectin levels were measured by ELISA analysis using Enzyme Immunoassay Kit (Boster, Inc. Wuhan, China) according to the instructions of the supplier.

### Nuclear translocation of NF-κB

Nuclear translocation of NF-κB was determined by western blot with RelA (p65)-specific antibody (Cell Signaling Technology, Danvers, MA, USA) as previously described^44^. Nuclear and cytoplasmic proteins were extracted with NE-PER^®^ Nuclear and Cytoplasmic Extraction Reagents according to the manufacturer’s instructions (Thermo Scientific, Waltham, MA, USA). Briefly, HUVECs were harvested with trypsin, then centrifuged at 500 × *g* for 5 min. The cell pellet was washed and resuspended with PBS. After carefully removing the supernatant, 100 µl ice-cold CERI containing protease inhibitors was added into the pellet, then the tube was vortexed vigorously for 15 s to resuspend the cell pellet thoroughly and incubated on ice for 10 min. After that 11 µl ice-cold CERII was added to the tube and vortexed vigorously for 5 s and incubated on ice for 1 min. The tube was vortexed for another 5 s and centrifuged at 16,000 × *g* for 5 min. The supernatant was the cytoplasmic protein. The pellet which contained nuclei was resuspended with 50 µl ice-cold NER containing protease inhibitors, vortexed vigorously for 15 s and incubated on ice, vortexed the tube 15 s for every 10 min 4 times. At last the tube was centrifuged at 16,000 ×*g* for 10 min and the supernatant (nuclear protein) was transferred into a ice-cold tube.

### Statistical analysis

All data are presented as mean ± SEM. Statistical analysis was determined by an unpaired two-tailed Student t test or one-way analysis of variance (ANOVA) followed by Bonferroni’s multiple comparison post hoc test with a 95% confidence interval. Values of *p* < 0.05 were considered significant.

## Supplementary information

Supplemental material
