# Supplementary material for: MicroRNA-181a-5p and microRNA-181a-3p cooperatively restrict vascular inflammation and atherosclerosis
Source: Cell Death Dis. 2019 May 7;10(5):365. doi: 10.1038/s41419-019-1599-9 (PMC6504957; doi:10.1038/s41419-019-1599-9)
Supplement: Supplementary file 1 — Supplemental material [file 41419_2019_1599_MOESM1_ESM.doc]

**Supplementary Tables and Figures**

**Supplementary Tables**

**Supplemental Table 1. Lipid and body weight parameters after miR-181a-5p mimics treatment**

|  | Cholesterol  (mg/dl) | Triglyceride  (mg/dl) | HDL  (mg/dl) | LDL  (mg/dl) | Body weight  (g) |
| --- | --- | --- | --- | --- | --- |
| ND | 434.5±12.1 | 135.6±4.7 | 18.1±0.8 | 93.6±11.0 | 24.9±2.1 |
| HFD | 1043.2±44.2 | 164.8±6.5 | 18.4±1.1 | 286.9±21.2 | 28.3±1.9 |
| HFD+NC-m | 1036.7±56.0 | 167.9±5.1 | 18.0±0.9 | 274.6±12.5 | 27.2±1.2 |
| HFD+181a-5p-m | 1038.9±40.1 | 167.1±7.0 | 18.5±1.2 | 284.1±10.4 | 28.2±2.0 |

Plasma cholesterol, triglyceride, HDL, LDL were obtained at sacrifice. All data are expressed as mean±SEM. ND, normal diet; HFD, high-fat diet; NC-m, negative control miRNA; 181a-5p-m, miR-181a-5p mimics; HDL, high density lipoprotein; LDL, low density lipoprotein. N=6 per group.

**Supplemental Table 2. Lipid and body weight parameters after miR-181a-3p mimics treatment**

|  | Cholesterol  (mg/dl) | Triglyceride  (mg/dl) | HDL  (mg/dl) | LDL  (mg/dl) | Body weight(g) |
| --- | --- | --- | --- | --- | --- |
| ND | 432.7±11.1 | 127.3±5.7 | 17.9±1.0 | 95.8±10.0 | 25.9±1.3 |
| HFD | 1016.6±46.5 | 160.4±8.5 | 18.9±1.3 | 279.2±22.2 | 29.3±1.0 |
| HFD+NC-m | 1028.3±53.0 | 165.3±7.9 | 18.3±1.7 | 281.6±11.5 | 28.2±2.1 |
| HFD+181a-3p-m | 1037.2±42.1 | 164.8±6.0 | 18.4±1.4 | 284.6±9.4 | 27.2±2.3 |

Plasma cholesterol, triglyceride, HDL, LDL were obtained at sacrifice. All data are expressed as mean±SEM. ND, normal diet; HFD, high-fat diet; NC-m, negative control miRNA; 181a-3p-m, miR-181a-3p mimics; HDL, high density lipoprotein; LDL, low density lipoprotein. N=6 per group.

**Supplemental Table 3. Primers used for RT-PCR analysis**

| Genes | primers | sequences |
| --- | --- | --- |
| *Vcam-1* | Forward | 5’- GTTCCAGCGAGGGTCTACC-3’ |
|  | Reverse | 5’- AACTCTTGGCAAACATTAGGTGT-3’ |
|  |  |  |
| *Icam1* | Forward | 5’- GTGATGCTCAGGTATCCATCCA-3’ |
|  | Reverse | 5’- CACAGTTCTCAAAGCACAGCG-3’ |
|  |  |  |
| *E-selectin* | Forward | 5’- ATGCCTCGCGCTTTCTCTC-3’ |
|  | Reverse | 5’- GTAGTCCCGCTGACAGTATGC-3’ |
|  |  |  |
| *Tnf-α* | Forward | 5’-TTCTGTCTACTGAACTTCGGGGTGATCGGTCC-3’ |
|  | Reverse | 5’-GTATGAGATAGCAAATCGGCTGACGGTGTGGG-3’ |
|  |  |  |
| *Il-1β* | Forward | 5’-ATGGCAACTGTTCCTGAACTCAACT-3’ |
|  | Reverse | 5’-CAGGACAGGTATAGATTCTTTCCTTT-3’ |
|  |  |  |
| *Il-6* | Forward | 5’-AGGATACCACTCCCAACAGACCT-3’ |
|  | Reverse | 5’-CAAGTGCATCATCGTTGTTCATAC-3’ |
|  |  |  |
| *Cxcl-2* | Forward | 5’-ACTTCAAGAACATCCAGAGCTTGA-3’ |
|  | Reverse | 5’-CTAGACTTCTGTCTGGGCGC-3’ |
|  |  |  |
| *18s rna* | Forward | 5'-CGGCTACCACATCCAAGGAA-3' |
|  | Reverse | 5'-CTGGAATTACCGCGGCT-3' |
|  |  |  |

**Supplementary Figures**

**Supplemental Figure 1**

**
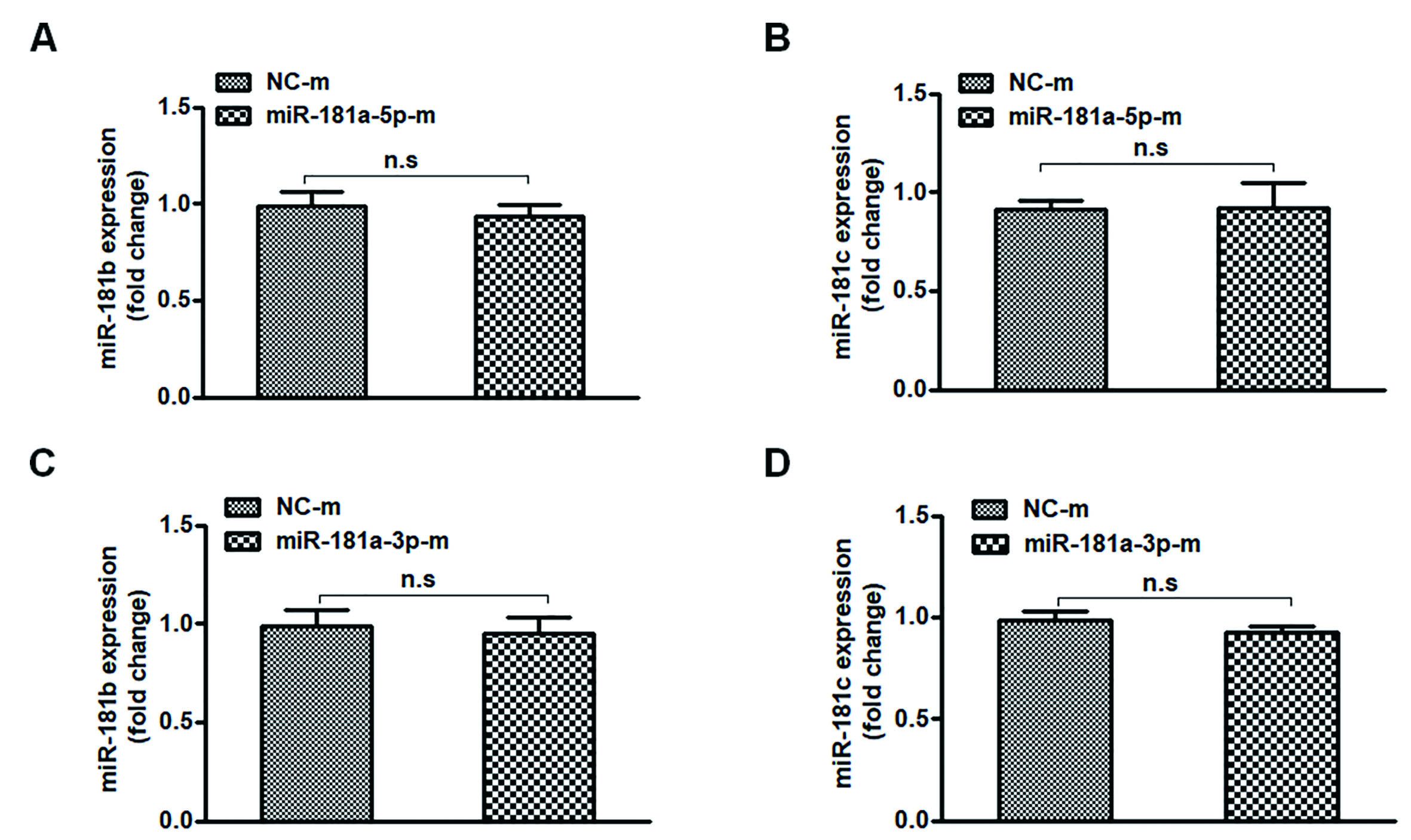
**

**Supplemental Figure 1.** **MiR-181b and miR-181c expression in ApoE^−/−^ mice injected with miR-181a mimics.**

ApoE^−/−^ mice were treated with 181a-5p-m, 181a-3p-m or NC-m, miR-181b (a and c) and miR-181c (b and d) expression were examined by quantitative polymerase chain reaction (qPCR). Data were shown by mean ± SEM (n=5).

**Supplemental Figure 2**

**
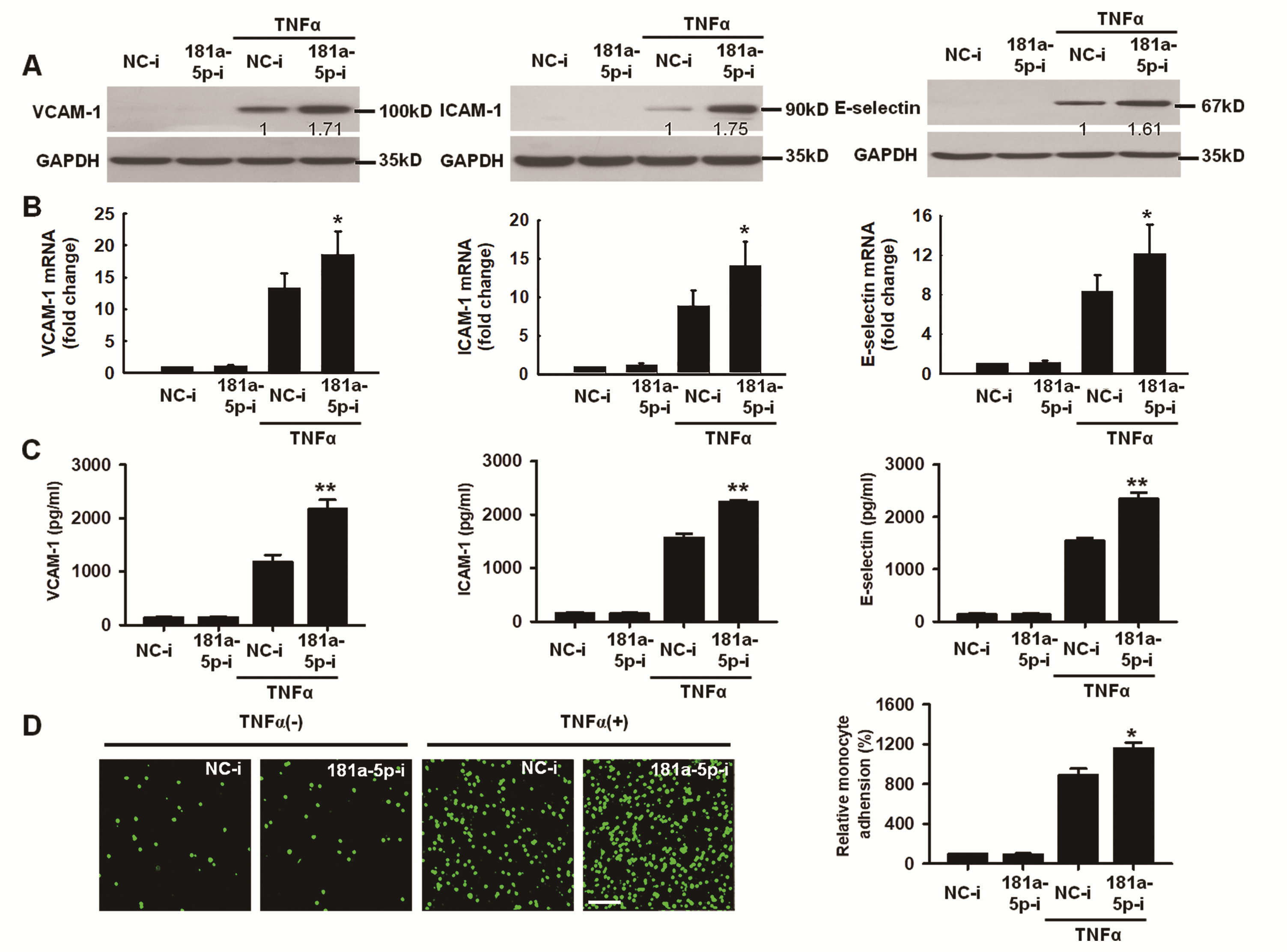
**

**Supplemental Figure 2. MiR-181a-5p** **inhibitor** **potentiates TNF-α–induced proinfammatory gene expression in HUVECs.**

a, representative western blotting of VCAM-1, ICAM-1 and E-selectin protein levels in HUVECs transfected with miRNA inhibitor negative control (NC-i) or miR-181a-5p inhibitor (181a-5p-i) after TNF-α (10 ng/ml) treatment for 12 hours (n=6 per group). b, Real-time qPCR analysis of VCAM-1, ICAM-1 and E-selectin mRNA levels in HUVECs treated with miRNA inhibitor negative control (NC-i) or miR-181a-5p inhibitor (181a-5p-i) after exposure to 10 ng/ml TNF-α for 4 hours (n=6 per group). c, HUVECs reveived miRNA inhibitor negative control (NC-i) or miR-181a-5p inhibitor (181a-5p-i) were exposed to TNF-α (10 ng/ml) for 24 hours. VCAM-1, ICAM-1 and E-selectin protein levels in cell culture medium were determined by ELISA analysis (n=5 per group). d, representative photo images and quantification of calcein-labeled THP-1 monocytes adhering to TNF-α-activated HUVECs transfected with miRNA inhibitor negative control (NC-i) or miR-181a-5p inhibitor (181a-5p-i) (n=6 per group). Values represent mean±SEM, scale bar: 100 um, *P < 0.05; **P < 0.01 vs. TNF-α+NC-i.

**Supplemental Figure 3**

**
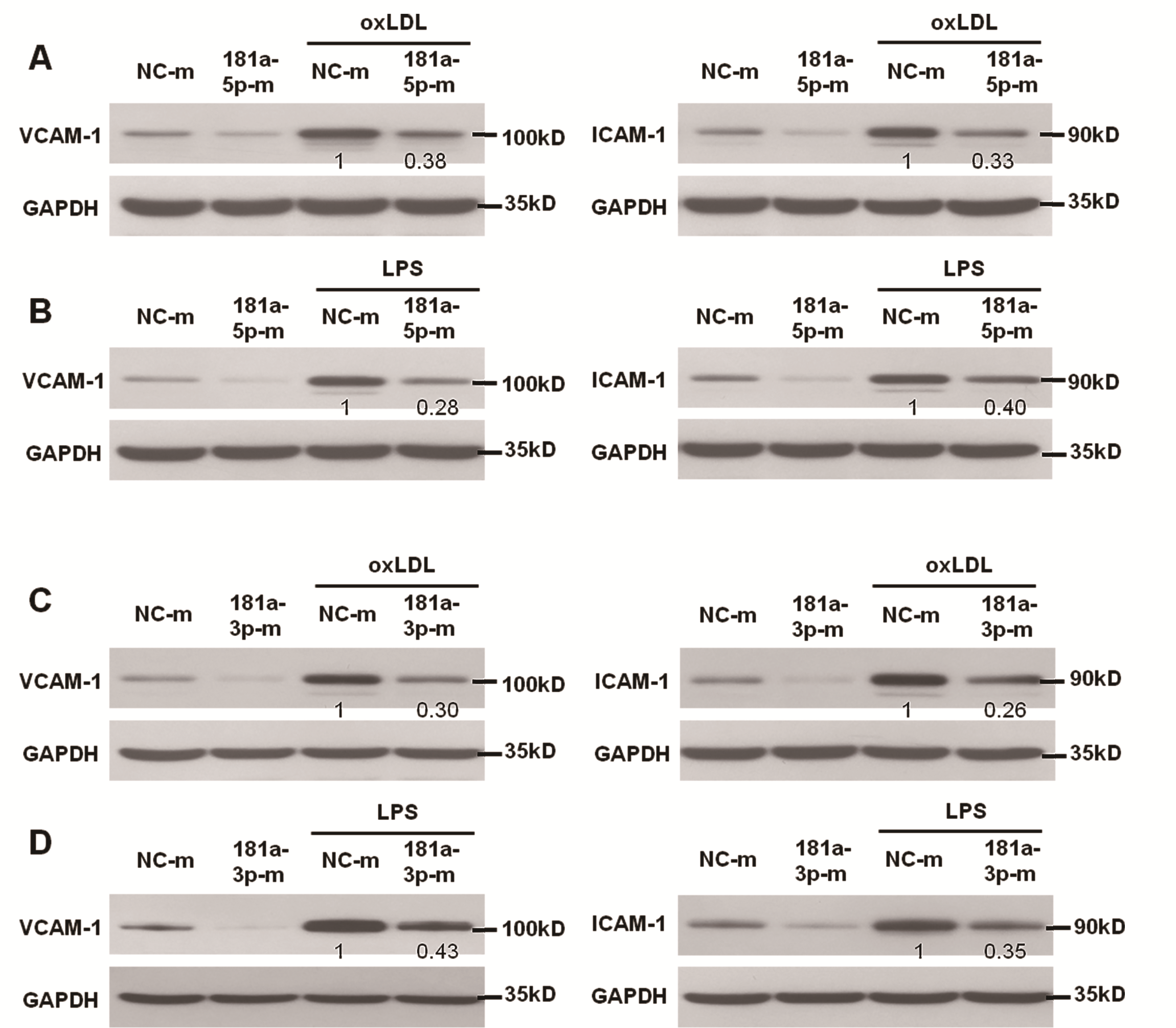
**

**Supplemental Figure 3. MiR-181a-5p and miR-181a-3p block oxLDL- and LPS-induced adhesion molecule expression in HUVECs.**

a and b, representative western blot of VCAM-1 and ICAM-1 protein expression from 5 independent experiments in HUVECs transfected with nonspecific control miRNA (NC-m) or miR-181a-5p mimics (181a-5p-m) after 50 μg/ml oxLDL (a) or 1 μg/ml LPS (b) treatment for 12 hours. c and d, western blot analysis of VCAM-1 and ICAM-1 protein expression in HUVECs transfected with nonspecific control miRNA (NC-m) or miR-181a-3p mimics (181a-3p-m) followed by 50 μg/ml oxLDL (c) or 1 μg/ml LPS (d) treatment for 12 hours. N=4.

**Supplemental Figure 4**

**
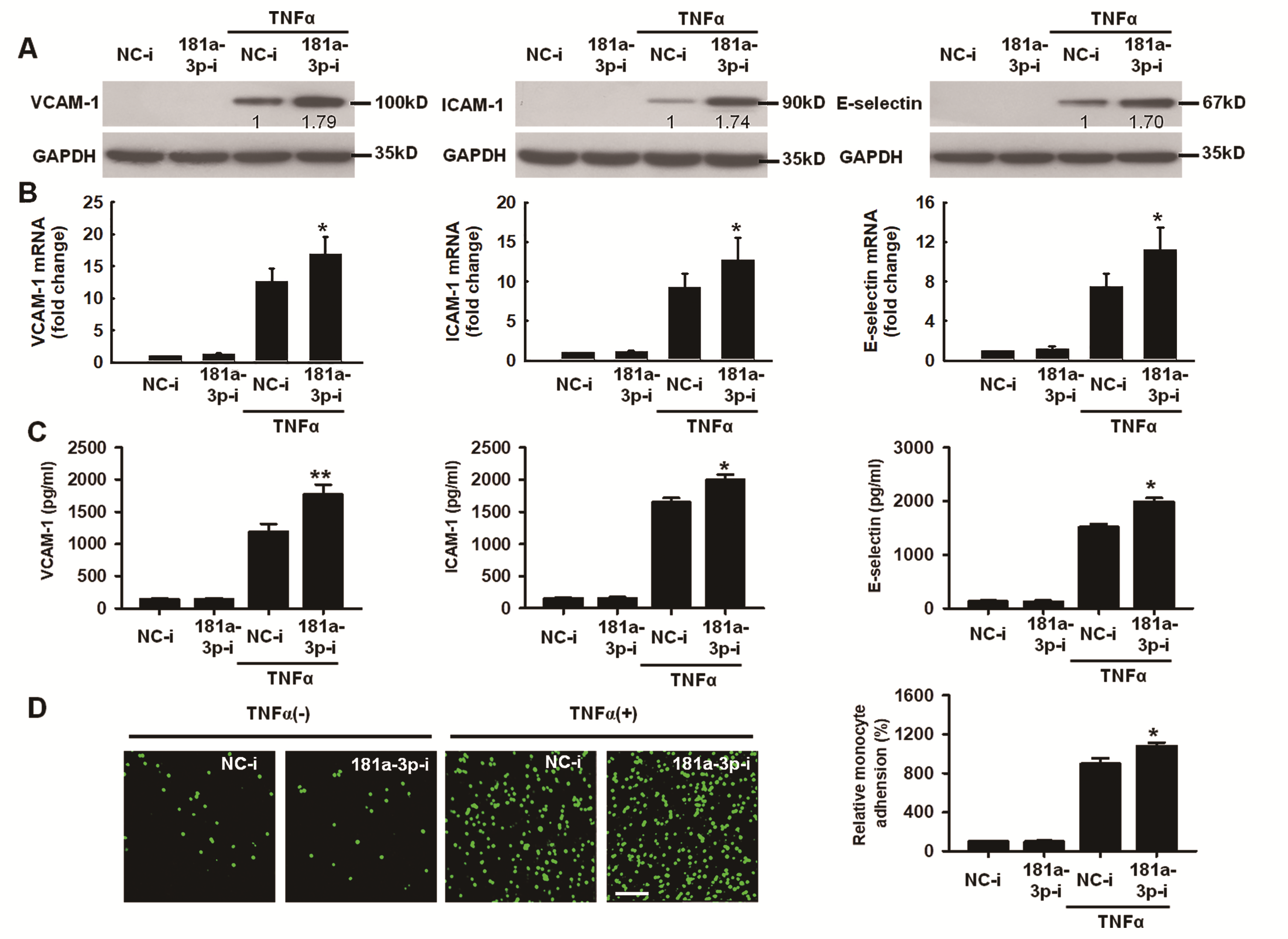
**

**Supplemental Figure 4. MiR-181a-3p** **inhibitor** **enhances TNF-α–induced proinfammatory gene expression in HUVECs.**

a, representative western blotting of VCAM-1, ICAM-1 and E-selectin protein levels in HUVECs transfected with miRNA inhibitor negative control (NC-i) or miR-181a-3p inhibitor (181a-3p-i) after TNF-α (10 ng/ml) treatment for 12 hours (n=6 per group). b, Real-time qPCR analysis of VCAM-1, ICAM-1 and E-selectin mRNA levels in HUVECs treated with miRNA inhibitor negative control (NC-i) or miR-181a-3p inhibitor (181a-3p-i) after exposure to 10 ng/ml TNF-α for 4 hours (n=5 per group). c, HUVECs reveived miRNA inhibitor negative control (NC-i) or miR-181a-3p inhibitor (181a-3p-i) were exposed to TNF-α (10 ng/ml) for 24 hours. VCAM-1, ICAM-1 and E-selectin protein levels in cell culture medium were determined by ELISA analysis (n=5 per group). d, representative photo images and quantification of calcein-labeled THP-1 monocytes adhering to TNF-α-activated HUVECs transfected with miRNA inhibitor negative control (NC-i) or miR-181a-3p inhibitor (181a-3p-i) (n=6 per group). Values represent mean±SEM, scale bar: 50um, *P < 0.05; **P < 0.01 vs. TNF-α+NC-i.

**Supplemental Figure 5**

**
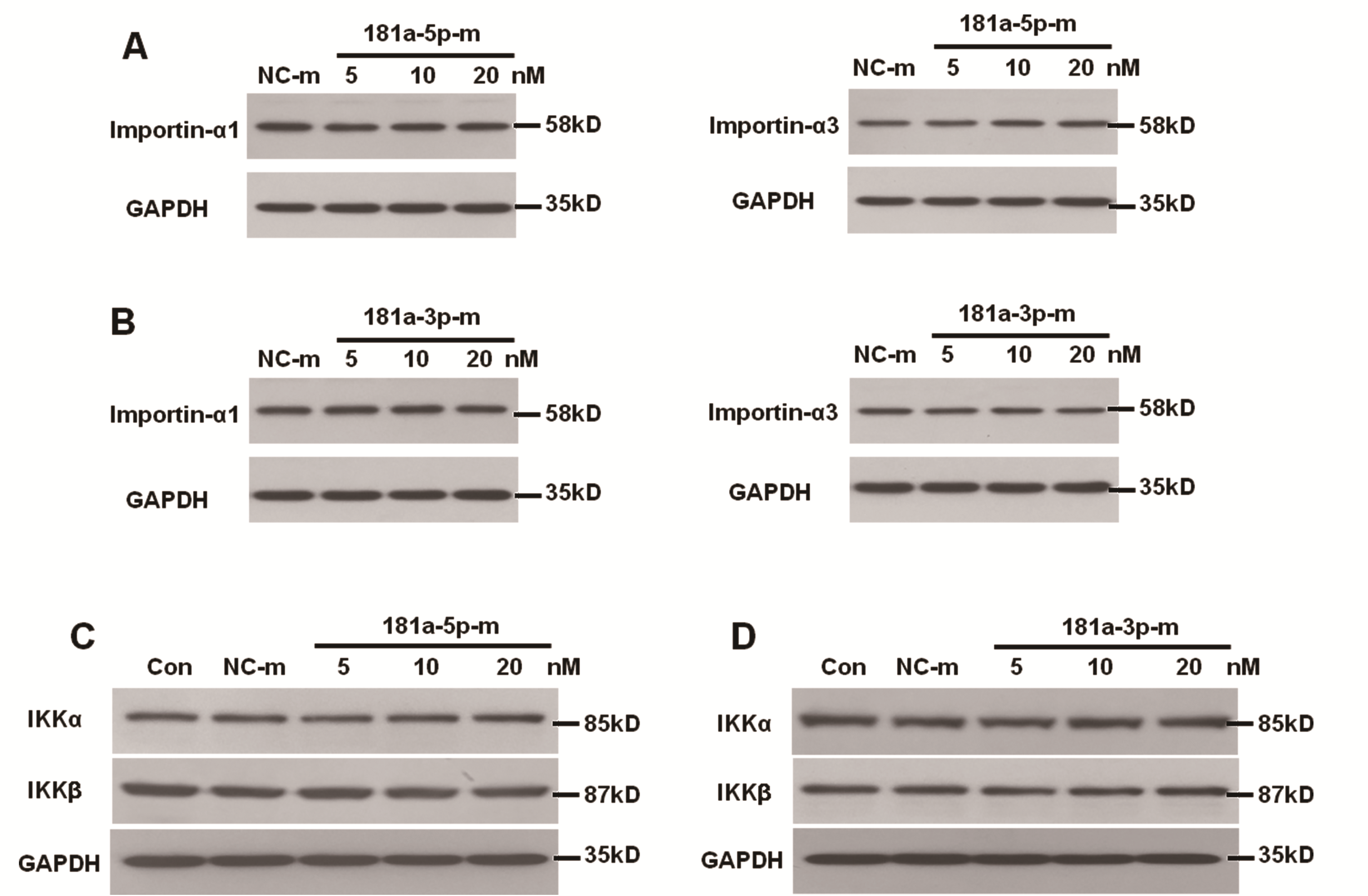
**

**Supplemental Figure 5. MiR-181a-5p and miR-181a-3p have no effects on importin-α1, importin-α3, IKKα and IKKβ protein expression in HUVECs.**

a, representative western blot of importin-α1 and importin-α3 protein expression from 4 independent experiments in HUVECs transfected with nonspecific control miRNA (NC-m) or miR-181a-5p mimics (181a-5p-m). b, representative western blot of importin-α1 and importin-α3 protein expression from 4 independent experiments in HUVECs transfected with nonspecific control miRNA (NC-m) or miR-181a-3p mimics (181a-3p-m). c, western blot analysis of IKKα and IKKβ protein expression in HUVECs transfected with nonspecific control miRNA (NC-m) or miR-181a-3p mimics (181a-3p-m) (n=5). d, western blot analysis of IKKα and IKKβ protein expression in HUVECs transfected with nonspecific control miRNA (NC-m) or miR-181a-5p mimics (181a-5p-m) (n=4).

**Supplemental Figure 6**


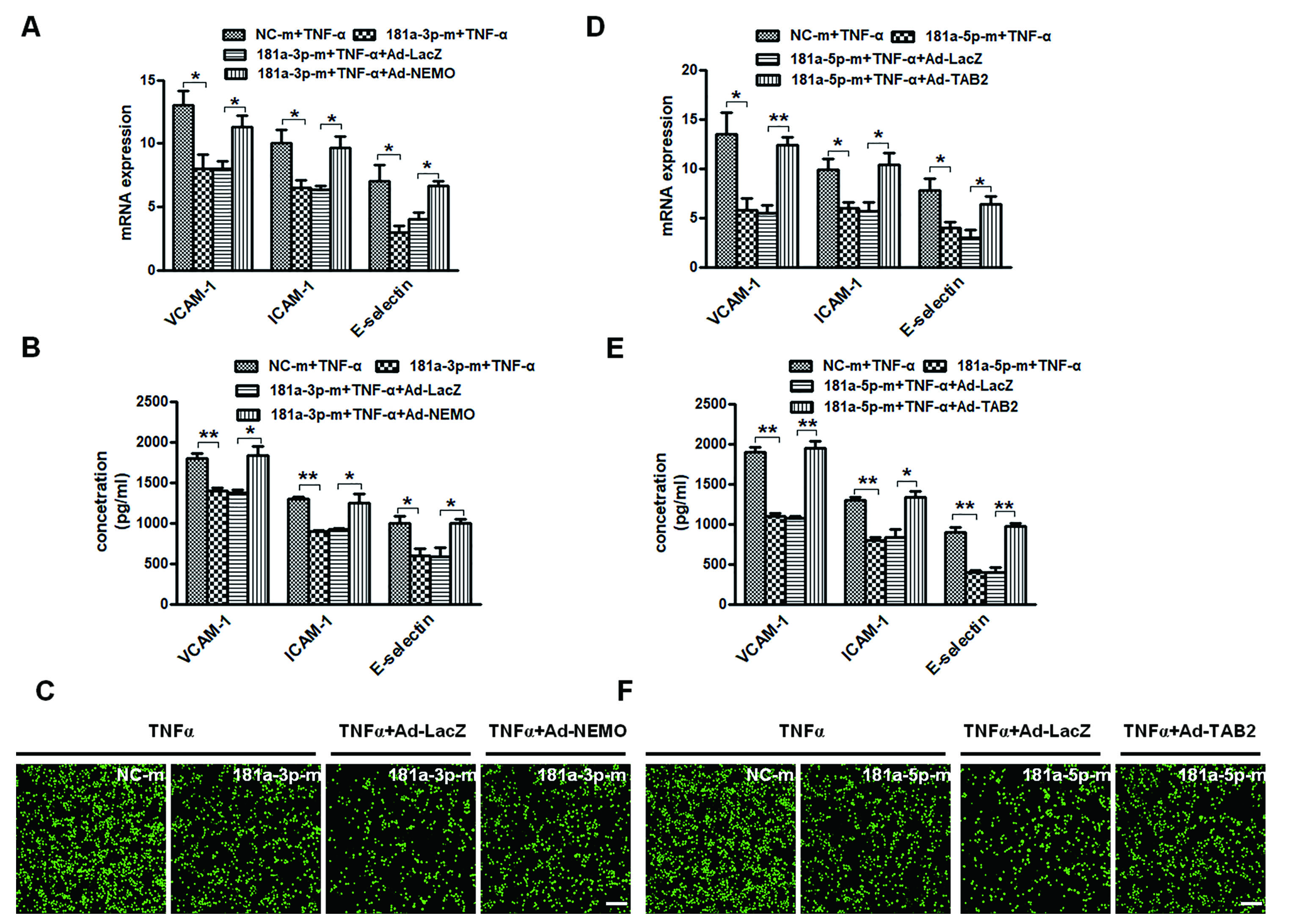


**Supplemental Figure 6**. **MiR-181a-3p targets NEMO and miR-181a-5p targets TAB2.**

a to c, HUVECs were infected with Ad-NEMO or Ad-LacZ, the effects of 181a-3p-m or NC-m on TNF-α induced mRNA expression of VCAM-1, ICAM-1 and E-selectin (a) , the concentration of soluble VCAM-1, ICAM-1 and E-selectin in the culture medium (b) and the adhesion of of monocytes to HUVECs (c) were examined. d to f, HUVECs were infected with Ad-TAB2 or Ad-LacZ, the effects of 181a-5p-m or NC-m on TNF-α induced mRNA expression of VCAM-1, ICAM-1 and E-selectin (d) , the concentration of soluble VCAM-1, ICAM-1 and E-selectin in the culture medium (e) and the adhesion of of monocytes to HUVECs (f) were detected. Scale bar : 200 um. *P < 0.05; **P < 0.01.

**Supplemental Figure 7**

**
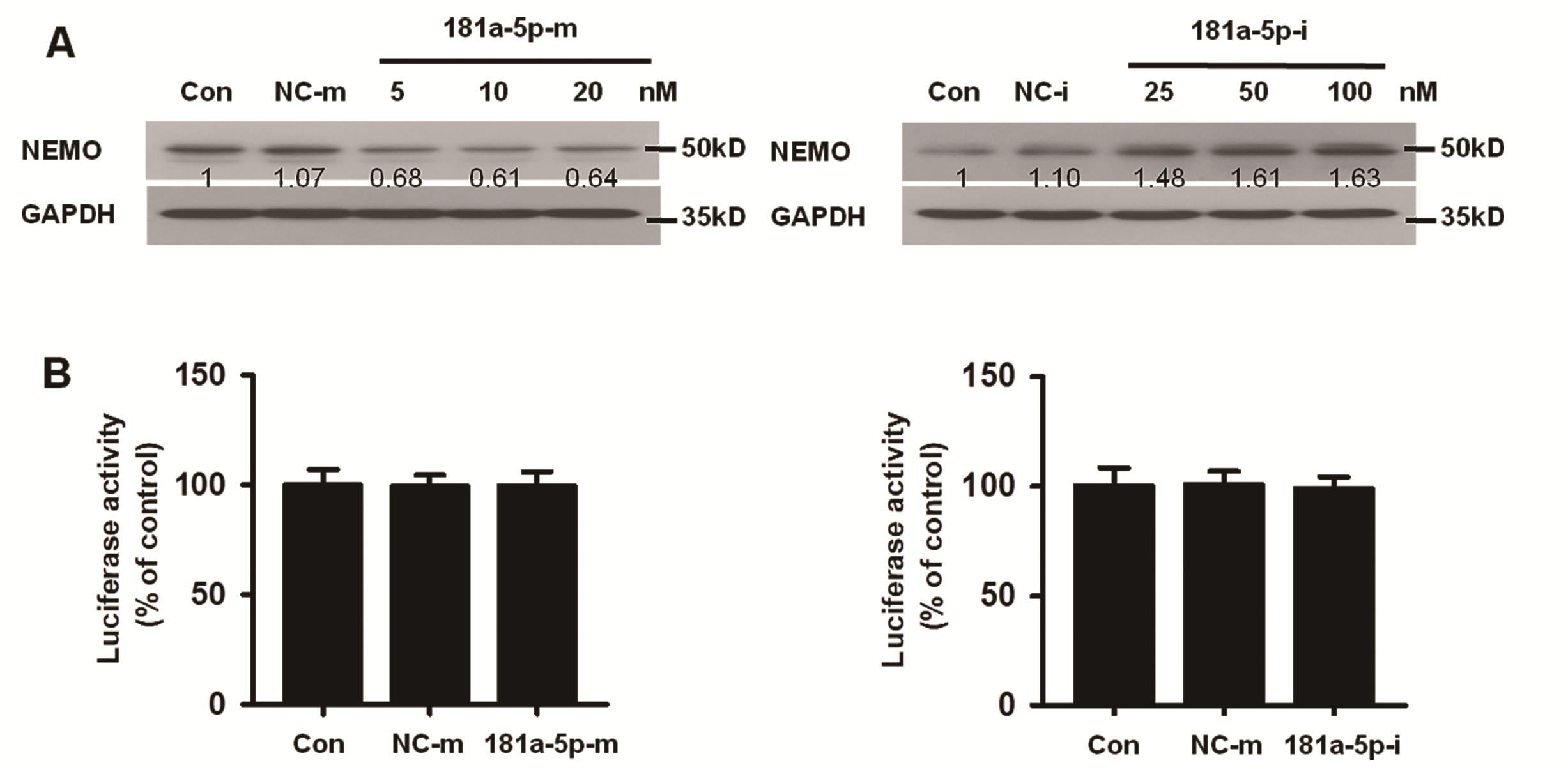
**

**Supplemental Figure 7. MiR-181a-5p** **regulates NEMO expression indirectly.**

a, western blotting of NEMO protein expression in HUVECs transfected with nonspecific control miRNA (NC-m) or miR-181a-5p mimics (181a-5p-m), miRNA inhibitor negative control (NC-i) or miR-181a-5p inhibitor (181a-5p-i). N=6. b, Luciferase reporter constructs containing 3’-UTR (Luc-NEMO-3’-UTR) was cotransfected with nonspecific control miRNA (NC-m) or miR-181a-5p mimics (181a-5p-m), miRNA inhibitor negative control (NC-i) or miR-181a-5p inhibitor (181a-5p-i) and the luciferase activities were assayed. N=5 per group.

**Supplemental Figure 8**

**
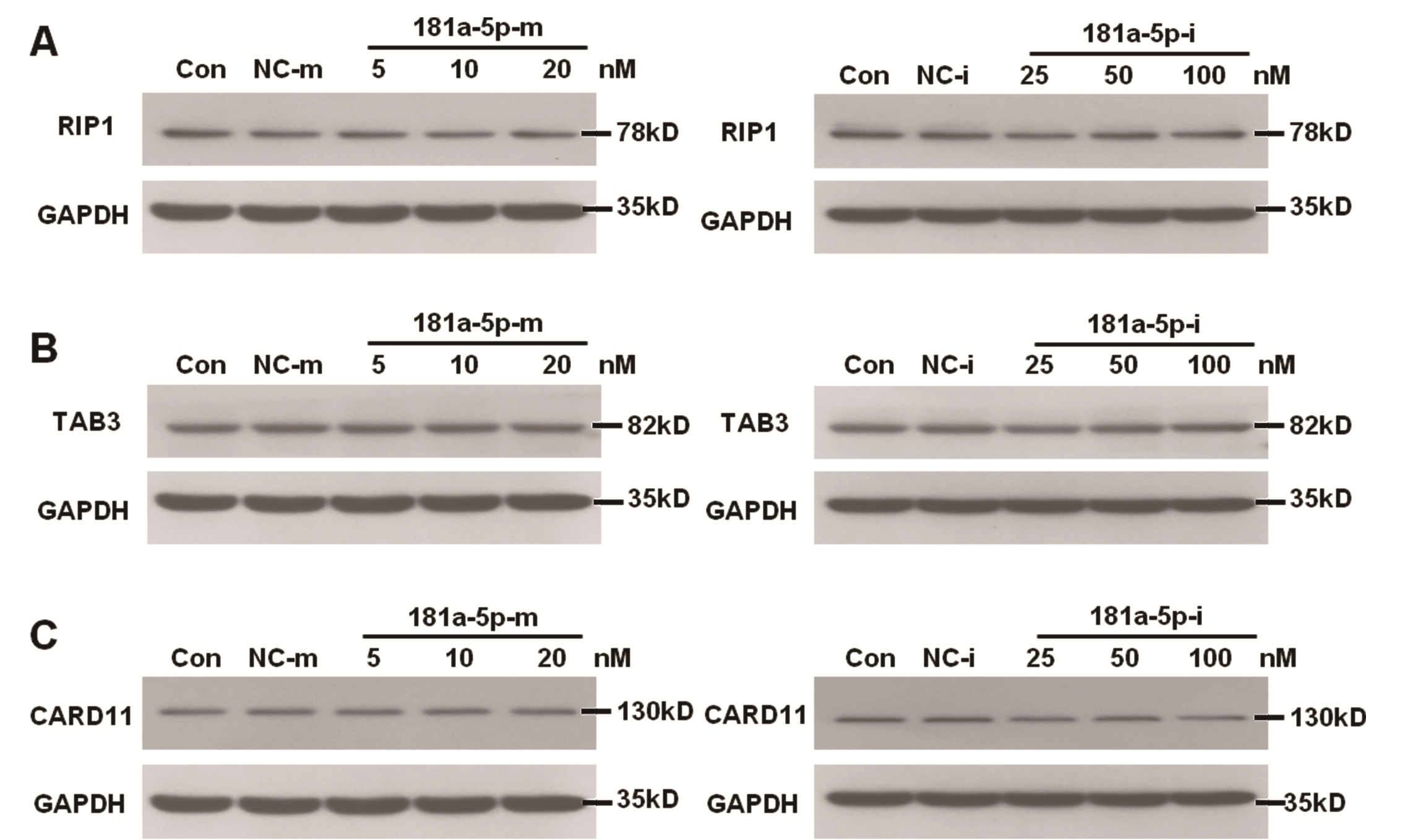
**

**Supplemental Figure 8. RIP1, TAB3 and CARD11 are not the targets of miR-181a-5p.**

Representative western blotting from 4-6 independent experiments of RIP1(a), TAB3 (b) and CARD11 (c) protein expression in HUVECs transfected with nonspecific control miRNA (NC-m) or miR-181a-5p mimics (181a-5p-m), miRNA inhibitor negative control (NC-i) or miR-181a-5p inhibitor (181a-5p-i).
